# Sensory innervation in the prostate and a role for calcitonin gene-related peptide in prostatic epithelial proliferation

**DOI:** 10.3389/fnmol.2024.1497735

**Published:** 2024-12-18

**Authors:** Hanyu Xia, Travis J. Jerde, Jill C. Fehrenbacher

**Affiliations:** ^1^Department of Pharmacology and Toxicology, Indiana University School of Medicine, Indianapolis, IN, United States; ^2^Indiana University Melvin and Bren Simon Comprehensive Cancer Center, Indiana University School of Medicine, Indianapolis, IN, United States; ^3^Stark Neurosciences Research Institute, Indiana University School of Medicine, Indianapolis, IN, United States; ^4^Indiana Center for Musculoskeletal Health, Indiana University School of Medicine, Indianapolis, IN, United States

**Keywords:** prostate, prostate innervation, sensory nerves, calcitonin gene-related peptide, tissue clearing, 3D imaging, cell proliferation, deep learning image segmentation

## Abstract

**Introduction:**

The prostate is densely innervated like many visceral organs and glands. However, studies to date have focused on sympathetic and parasympathetic nerves and little attention has been given to the presence or function of sensory nerves in the prostate. Recent studies have highlighted a role for sensory nerves beyond perception of noxious stimuli, as anterograde release of neuropeptides from sensory nerves can affect vascular tone and local immune responses.

**Methods:**

To identify the degree of sensory innervation in the prostate, we utilized state-of-the-art tissue clearing and microscopy to visualize sensory innervation in the different lobes of the mouse prostate. To determine whether sensory nerves have a role in regulating proliferation within the prostate, we used an intersectional genetic and toxin approach to ablate peptidergic sensory nerves systemically.

**Results:**

We found that sensory neurons are abundant in the prostate both in nerve bundles along the vasculature and as independent nerve fibers wrapped around prostatic acini in a net-like fashion. In addition to the dense innervation of the prostate, we found that *Calca* haploinsufficiency, the genotype control for our intersectional ablation model, results in a diminished level of Ki67 staining in the stromal compartment of the dorsal lobe and a diminishing Ki67 trend in other lobes.

**Discussion:**

These findings suggest that sensory neurons might have developmental or homeostatic effects within the prostate. Further studies are warranted to assess the role of sensory neurons and the sensory neuropeptides on prostatic development and on proliferation in the presence of pro-inflammatory stimuli such as bacterial infection or tumor cells.

## Introduction

The prostate is a male accessory sex organ that plays a key role in reproductive function by maintaining sperm quality and viability. The prostate is composed of small glandular epithelial structures, surrounded by a layer of smooth muscle and basement membrane known as acini, which are responsible for producing and secreting components of the seminal fluid. Prostate development in mammals begins midway during fetal development but pauses at birth and resumes during puberty when androgen levels rise significantly (Cunha et al., 2018). In addition to androgens, prostatic development is regulated by morphogens, growth factors and cytokines (Grishina et al., 2005; Luo et al., 2013; Thomson and Marker, 2006; Jerde and Bushman, 2009). During the highly coordinated process of prostate development, epithelial budding and subsequent branching morphogenesis into the stroma drive the formation of the prostate’s characteristic structure, while maintaining distinct stromal and epithelial compartmentalization (Cunha et al., 2018). In humans, this process leads to the formation of three histological zones—peripheral, central, and transition—while in mice, it develops into distinct lobes: anterior, dorsal, lateral, and ventral (McNeal, 1981; Ittmann, 2018; Cunha et al., 2018; Oliveira et al., 2016). Neuronal and vascular arborization accompany epithelial branching during development (Jen et al., 1995); however, the roles of specific neuronal subtypes during this process remain largely uncharacterized.

Two of the most common health concerns in urology occur in the prostate: prostate cancer and benign prostatic hyperplasia (BPH). Approximately 50% of men by the age of 50 and 90% of men over 80 have BPH (McVary, 2006), an aging-associated syndrome defined by lower urinary tract symptoms (LUTS) such as increased frequency, urgency, and often an enlarging prostate gland that can compress the urethra over time. This compression can lead to additional urinary issues, including difficulty initiating urination, weak urine flow, frequent urination, and a persistent feeling of incomplete bladder emptying (Fibbi et al., 2010). However, many patients with non-enlarged prostates are also symptomatic, suggesting that idiopathic neuronal involvement may contribute to these symptoms, emphasizing the importance of understanding the prostate’s neuronal makeup.

Prostate cancer is the second most prevalent male cancer and the fourth leading cause of cancer-related deaths among men worldwide, affecting approximately 14% of men and accounting for 4% of global cancer-related deaths (Bray et al., 2024). Historically regarded as a disease of older men, the age of diagnosis has steadily declined over the past decades, a trend that cannot be solely attributed to improved screening (Zhou et al., 2016). Despite its high incidence, most prostate cancers are indolent, contributing to a five-year survival rate of 97.5% (National Cancer Institute, 2024) leaving many patients to contend with the long-term side effects of cancer treatment. Prostate cancers grow locally in response to microenvironmental factors such as growth factors produced by stromal cells, cytokines released from inflammation, and vascular derived cues (Gal et al., 2017; Bahmad et al., 2021; Ge et al., 2022). Additionally, neuronal factors have been proposed to also promote tumor growth and invasion (Zahalka and Frenette, 2020). Understanding the types of nerves present and their role in prostate cancer progression could offer key insights and identify potential therapeutic targets.

Stromal-epithelial interactions are fundamental to prostate development (Cunha, 1972; Cunha et al., 1980; Toivanen and Shen, 2017), and growth factors, cytokines, and inflammatory mediators derived from the local microenvironment are known to influence prostate cancer progression and BPH development. The prostate pathologies described above highlight how local innervation of the glandular microenvironment plays a role in disease progression, particularly as patients age. For autonomic nerves, there have been clear studies to examine the innervation density of the prostate (Reeves et al., 2019; Magnon et al., 2013; Hu et al., 2022; Turco et al., 2019; White et al., 2013) and functional studies to examine how autonomic nerve activity alters both healthy and diseased prostate tissue (Magnon et al., 2013; Hu et al., 2022; Zahalka et al., 2017). However, limited and conflicting studies exist regarding the degree of sensory innervation in healthy or diseased tissue (Blasko et al., 2023; Pennefather et al., 2000; Wegner et al., 2017; McVary et al., 1998).

Sensory nerves are known to detect noxious stimuli, but a lesser-known function is the anterograde release of neuropeptides, such as substance P (SP) and calcitonin gene-related peptide (CGRP), within the innervated peripheral tissue. The local release of neuropeptides, termed neurogenic inflammation, is well understood to regulate vascular permeability (Hua, 1986; Hallberg and Pernow, 1975; Lembeck and Holzer, 1979) and has been shown to affect immune cells and keratinocytes to promote wound healing and alter somatic and visceral inflammation (Kjartansson et al., 1987; Westerman et al., 1993; Yan et al., 2019; Duan et al., 2017; Brain, 1997; Chiu et al., 2012; Richardson and Vasko, 2002; Zhu et al., 2024). The extent to which sensory neurons can influence other tissues and cell types—either maintaining homeostasis or affecting tissue healing and disease progression, such as in cancer—is the subject of many current research studies.

Previous studies on sensory innervation in the prostate have been limited to two-dimensional tissue sections, which limits our ability to discern a structure–function relationship for the sensory nerves that innervate the prostate. A better understanding of the overall structure and distribution is essential to advancing our understanding of how sensory nerves impact both healthy and diseased prostate tissue. To that end, we used state-of-the-art tissue clearing and microscopy to visualize sensory innervation in the different lobes of the mouse prostate in aged mice. Next, we used an intersectional genetic and toxin approach to ablate peptidergic sensory nerves with diphtheria toxin (DTX) administration, originally designed by Dr. Mark Zylka (McCoy et al., 2013). In this model, novel human diphtheria toxin receptor (hDTR) expression is induced in peripheral nerves with promoter activity for CGRP (*Calca*), limiting our ablation to peptidergic sensory nerves to determine whether peptidergic nerves modulate prostate tissue homeostasis.

## Materials and methods

### Animals

**Figure 1:**
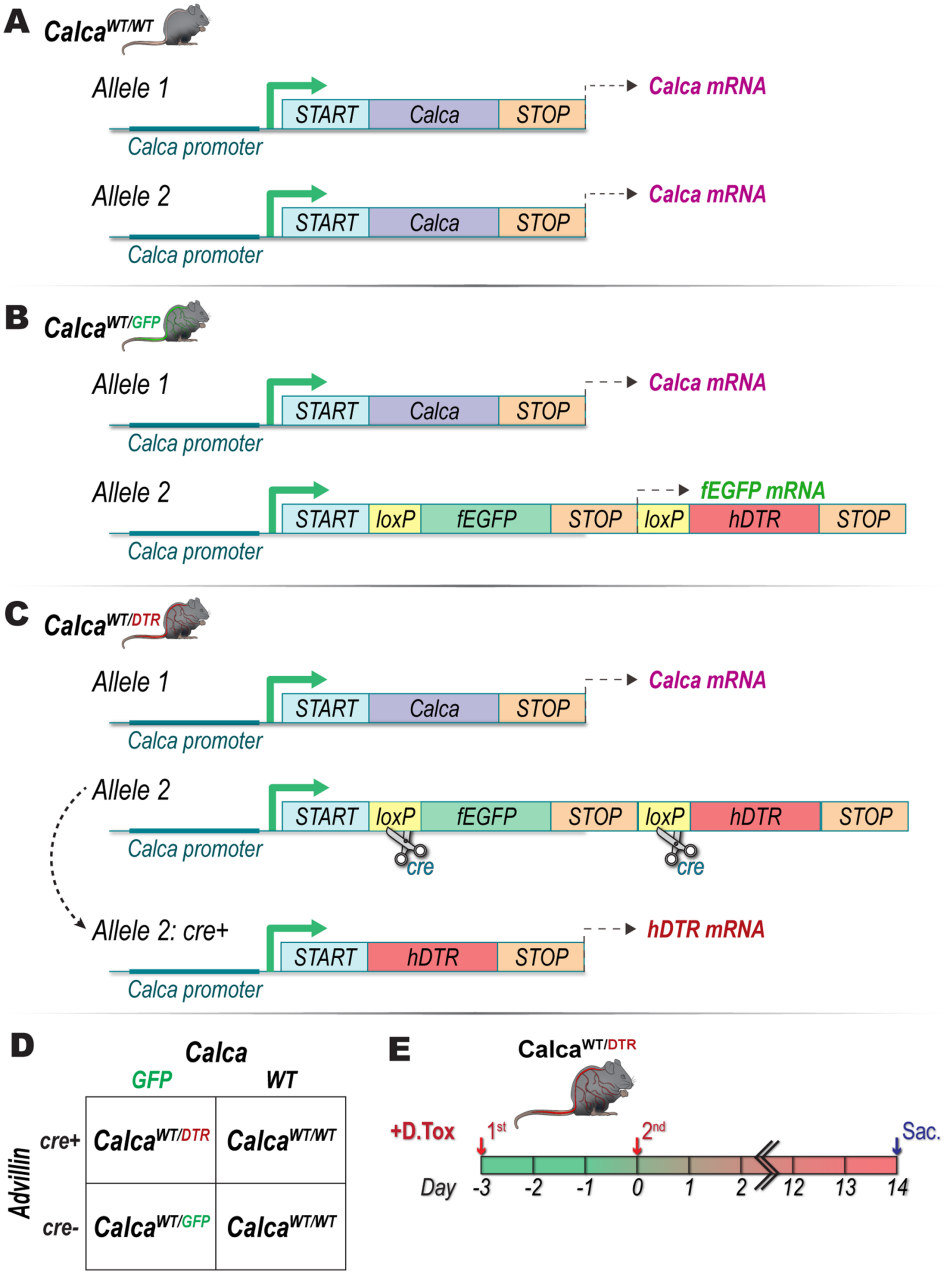
Genetic, breeding, and diphtheria toxin treatment schema. Genetic schema for **(A)** Calca^WT/WT^, **(B)** Calca^WT/GFP^, and **(C)** Calca^WT/DTR^ mice. **(D)** Male Advillin-Cre+/− mice were crossed with female Calca^WT/GFP^ mice to generate Calca^WT/DTR^ mice. **(E)** Calca^WT/DTR^ mice underwent two i.p. injections of DTX (100 μg/kg), 72 h apart before being sacrificed 14 days after second injection.

All animal experimental protocols were approved by the Institutional Animal Care and Use Committee at Indiana University School of Medicine, Indianapolis, IN and in compliance with the National Institutes of Health Guide for the Care and Use of Laboratory Animals. Animals were housed in group cages in a light-controlled room with food and water available *ad libitum*. Male Advillin-Cre^+/−^ animals were purchased from The Jackson Laboratory (strain #032536, Bay Harbor, ME, USA). Female, *Calca*-fEGFP^+/−^ [B6.129P2(Cg)—Calcatm1.1 (EGFP/HBEGF) Mjz/Mmnc, RRID: MMRRC_036773-UNC] animals for breeding, here on referred to as Calca^WT/GFP^ (Figure 1B), were a generous gift from the laboratory of Fletcher White, originally developed by the Mark Zylka lab at the University of North Carolina at Chapel Hill. Male, *Advillin-Cre^+/−^*, mice were crossed with female, Calca^WT/GFP^ (Figure 1D), mice to generate Calca^WT/GFP^, Calca^WT/DTR^ (Figure 1C), or wildtype Calca^WT/WT^ (Figure 1A) offspring. Diphtheria toxin (DTX; List Biological Laboratories, Product #150) was reconstituted to 20 μg/mL in sterile 0.9% saline, aliquoted, and stored at −80°C until used for injections. Once animals reached 10–12 months of age, DTX was administered by i.p. injection (100 μg/kg) twice with 72 h between the two injections. Animals were sacrificed and prostate tissue was harvested 14 days after the second injection (Figure 1E).

### Thermal sensitivity testing

Thermal sensitivity testing was performed 14 days after the second DTX injection to confirm sensory ablation before sacrifice. Hargreaves test (Ugo Basile, catalog number: 37370) was performed on *Calca^WT/DTR^*, Calca^WT/GFP^, and wildtype mice 14 days after the second DTX injection to assess thermal sensitivity. Animals were allowed to acclimate to the testing chamber for 1 h before the start of testing. The infrared intensity was set to 30, and the emitter was positioned directly underneath the center of the left or right hindpaw. A minimum of 5 trials were conducted on each animal with a minimum of 5 min between each trial. Times to elicit a withdrawal response to thermal heat were recorded for each animal.

### Tissue processing

Mouse prostate was harvested from animals and fixed in 4% (v/v) paraformaldehyde (PFA) in PBS overnight at 4°C with gentle rocking. The next day, fixed prostates were then washed three times with PBS for 30 min each followed microdissection of the prostate. The bladder, seminal vesicles, and excess adventitia were carefully removed to allow for visualization of the midline and urethra whereby a midsagittal bisection was performed to separate the prostate into two halves. Half of the prostate was embedded in paraffin, and the other halves were further microdissected to isolate individual prostate lobes, while taking care to preserve points of attachment to the urethra in order to preserve tissue orientation. The individual lobes were then processed for tissue clearing with immunolabeling. Photomicrographs of the whole prostate and prostate lobes were acquired under a stereo dissection microscope (Nikon, SMZ460).

### Tissue clearing, immunofluorescence labeling and 3D imaging

Optical tissue clearing was performed through a modified ethyl-cinnamate based technique (Masselink et al., 2019; Huang et al., 2019). Fixed tissues were washed with PBS and decolorization was performed with 25% (w/w) Quadrol (N′-Tetrakis(2-Hydroxypropyl)ethylenediamine), 15% (w/w) Triton-X100, and ddH_2_O for 48 h at 37°C. Tissues were permeabilized with 2% Triton-X100, 20% DMSO, 5% BSA, 0.05% NaN, in PBS (PBS-TxDBN) for 48 h at room temperature followed by immunolabeling with primary and secondary antibodies in 5% normal donkey serum PBS-TxDBN for 48 h at 37°C. Labeled tissue was then incubated overnight with 4% PFA:PBS at 4°C and washed with PBS. Nuclear staining was performed with DAPI (1:1000, Thermo Scientific, 62248) for 24 h at room temperature. Graded dehydration was performed with 30, 50, 70, 100% ethanol in ddH_2_O. After dehydration, tissues were cleared by immersion in 100% ethyl cinnamate for refractory index matching. Cleared and immunolabeled samples were stored in ethyl cinnamate in 5 mL tubes until imaged by confocal microscopy on a Leica TCS SP8 DIVE with the HC PL APO 20x/0,75 IMM CORR CS2 objective (Leica Microsystems, 11506343). Image stacks were captured at 400 Hz with 2-line average. To isolate discrete high-intensity puncta, indicative of antibody precipitates or debris, post-processing included dividing the images by a 3D mean sphere filter applied to the z-stack. The filtered image was then subtracted from the original image, and median background subtraction was performed using FIJI/ImageJ with CLIJ2 (Haase et al., 2020; Schindelin et al., 2012). 3D visualizations were created with Fluorender (Wan et al., 2017).

### 2D immunofluorescence

Fixed prostates underwent a gradual gradient ethanol dehydration to xylene before being embedded in paraffin blocks. Embedded tissues were sectioned on a rotary microtome (Leica HistoCore BIOCUT, 149BIO000C1), floated in a hot water bath, and adhered to glass slides (Fisherbrand™ Superfrost™ Plus, 12-550-15) for 2D immunofluorescence. Slide sections were baked in a hybridization oven to remove excess paraffin for 2 h and were then immersed in xylenes to remove any residual paraffin. Slides were then gradually rehydrated through an ethanol gradient. Plastic slide containers with retrieval buffer (10 mM Tris-base, 25 mM NaCl, and 0.05% Tween20; pH 10.0) were allowed to heat up to 95°C in a hot water bath. After deparaffinization and rehydration, slides were transferred to the containers in the water bath and immersed in heated retrieval buffer for 10 min. Next, the containers and slides were removed from the water bath and allowed to cool on the benchtop for 10 min before the slides were removed, submerged in ddH_2_O for 10 min, and continued onto immunofluorescence labeling. After epitope retrieval, tissue sections were blocked with 5% normal donkey serum and 0.05% Tween-20 in PBS. Sections were immunolabeled with primary antibodies at 4°C overnight, followed by three washes with PBS-Tween. The primary antibodies used for these studies were acquired from commercial vendors, except for the CGRP antibody, which was a generous gift from Michael Iadarola (NIH). Following washing, slides were incubated with donkey secondary antibodies conjugated to AlexaFluors at 4°C overnight. Primary and secondary antibody catalog numbers and concentrations used are indicated in Table 1. After incubation, sections were washed with PBS-Tween and stained with DAPI (1:1000; Thermo Scientific, 62248) or 20 μg/mL Hoechst 33342 for 10 min, followed by three additional washes with PBS-Tween. Hoechst was used for initial experiments, and later experiments utilized DAPI because it was empirically determined to be more amenable for 3D imaging studies. The sections were then mounted with Vectashield (Vector Laboratories, H-1700).

**Table 1:** Primary and secondary antibodies.

Use	Antibody	Conc.	Mfrs.	Cat.	RRID
Primary antibodies					
2D	Rb-CK5	1:300	Invitrogen	MA5-16372	AB_2537891
2D	Ms-CK 8	1:100	Novus Biologicals	NB120-9287	AB_921847
2D	Rt-Ki-67	1:100	Invitrogen	14–5,698-82	AB_10854564
2D	Ms-PanCK	1:100	Cell Signaling Technology	4,545	AB_490860
3D	Ms-PanCK	1:100	Invitrogen	MA518156	AB_2539530
3D	Gt-CD31/PECAM-1	1:300	R and D Systems	AF3628	AB_2161028
3D	Rb-TUBB3	1:100	Cell Signaling Technology	5,568	AB_10694505
2D, 3D	Ck-PGP9.5	1:500	Invitrogen	PA1-10011	AB_1088162
2D, 3D	Rb-CGRP	1:1000	MJ Iadarola [40]	–	–
Secondary antibodies					
2D	DkαMs-Alexa 488	1:200	Invitrogen	A21202	AB_141607
2D	DkαRt-Alexa 647	1:200	Invitrogen	A21247	AB_141778
3D	DkαGt-Alexa 568	1:200	Invitrogen	A11057	AB_2534104
2D, 3D	DkαRb-Alexa 488	1:200	Invitrogen	A32790	AB_2762833
2D, 3D	DkαCk-Alexa 647	1:200	Jackson ImmunoResearch Laboratories Inc.	703-605-155	AB_2340379

### Image analysis (2D)

Whole slide imaging was performed on 5 μm sections of formalin-fixed, paraffin-embedded (FFPE) mouse prostate tissue using the Akoya PhenoCycler-Fusion 2.0 system (Akoya Biosciences, Inc.). The imaging system was equipped with a Sony IMX421-based camera and the XCite MultiBand LED illumination system controlled by Fusion 2.1.0 software. A 20× objective lens was utilized for fluorescence image acquisition across five channels corresponding to DAPI (15 ms exposure, nuclei), Opal 520 (17.4 ms exposure, CK8), Opal 620 (220 ms, CK5), Opal 690 (100 ms, Ki67), and sample autofluorescence. Stitching was performed by the bulti-in software and generated a qptiff file with a pixel resolution of approximately 2.01 pixels per micron. Image visualization and analysis was conducted using QuPath software (Bankhead et al., 2017).

Each prostate section was manually annotated based on epithelial histomorphology and fluorescent signal for DAPI, CK5, and CK8, to designate the lobes—anterior, dorsal, lateral, and ventral—and the lumen (Supplementary Figure 1A). In order to analyze distinct anatomical regions within the prostate tissue, a binary pixel classifier was trained with 16 representative images that were manually annotated for the epithelium of each prostate lobes (512 × 512 pixels, four image per lobe). The pixel classifier used an input resolution of 0.4987 μm per pixel, input size of 512 × 512 pixels, and three input channels, DAPI, CK5, and CK8. Output type was set to probability with two channels (“Epithelium” and “non-epithelium”). The classifier’s operation pipeline included local normalization and multi-scale filtering operations at various scales. The pixel classifier was applied to each lobe annotation to generate preliminary epithelial compartment annotations. Preliminary compartment annotations were then smoothed and further manually refined as needed (Supplementary Figure 1B).

To detect, classify, and quantify proliferative cells, we used Cellpose (Stringer et al., 2021) integrated within QuPath via the ‘QuPath Cellpose Extension’ (Burri et al., 2024). The cellpose nuclei model was fine-tuned on 28 images of 500 × 500 pixels, extracted from random regions across the whole slide images obtained from this study. To refine the classification of the 1.028 M detections identified across the analyzed dataset, object classifiers were trained in QuPath using images masked for DAPI or Ki67. Features were added to detect cell objects which included morphological features and fluorescence signal intensity and texture. Intensity features for autofluorescence at the same resolution were also included, evaluated within circular regions of varying diameters. Random Trees classifier parameters were established as: maximum depth of 25, minimum sample count of 10, and 50 trees, handling up to 10 categories. The trained classifier was applied to detected cells to enhance accuracy in identifying DAPI and/or Ki67 labeled nuclei (Supplementary Figures 1C,D).

### Statistical analyses

Thermal sensitivity data were analyzed by one-way ANOVA with Tukey–Kramer HSD multiple comparisons test. The ratios of Ki67+ cells to DAPI+ cells in prostatic tissue sections were analyzed by one-way ANOVA with Dunnett’s multiple comparisons test comparing the Calca^WT/GFP^ and Calca^WT/DTR^ groups to wildtype. Graphs were generated and statistical tests were performed in GraphPad Prism 6.

## Results

### 3D visualization of prostate nerve fibers

**Figure 2:**
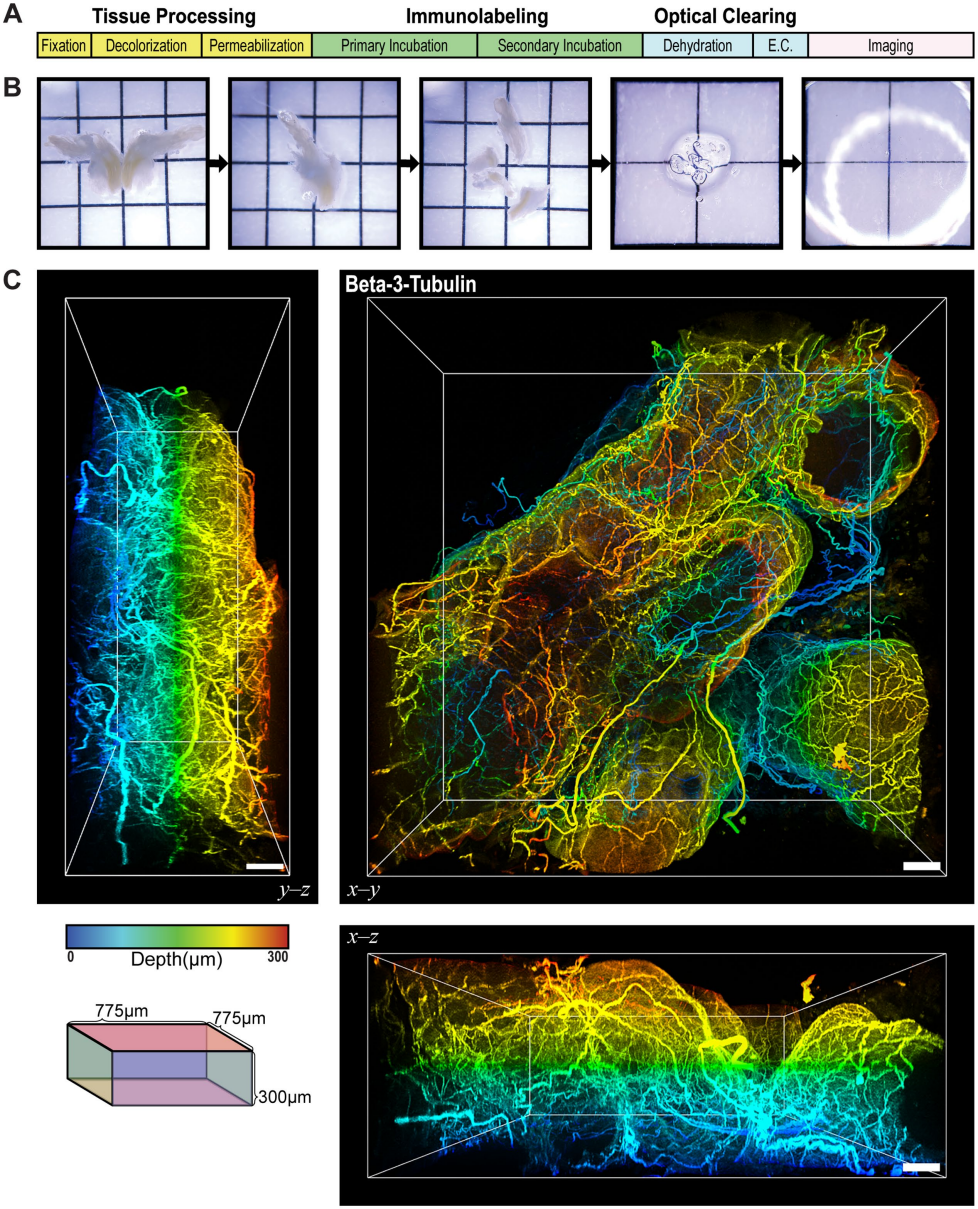
Ethyl Cinnamate-Based Optical Tissue Clearing Reveals Extensive Continuous Nerve Fibers in the Prostate. **(A)** A schematic of the general tissue processing workflow for ethyl cinnamate-based optical tissue clearing. The workflow begins with tissue processing, which includes fixation, decolorization, and permeabilization to prepare the tissue for antibody labeling. The tissues are immunolabeled with primary and secondary antibodies, dehydrated and immersed in ethyl cinnamate to optically clear the tissue. Finally, the cleared tissue is imaged using a confocal microscope. **(B)** Photomicrographs of the mouse prostate after microdissection and processing for tissue clearing. From left to right, the images show a whole bisected prostate, half of a prostate, the microdissected lobes, a single lobe after labeling and clearing, and a fully cleared lobe immersed in ethyl cinnamate, rendering it virtually transparent. **(C)** 3D immunofluorescence labeling of beta-3-tubulin in the ventral prostate of the mouse. Orthogonal views are presented in the left (Y-Z) and bottom (X-Z) panels. Scale bars represent 50 µm. Colorimetric scale represents the depth of the tissue in microns. Cube illustrates the imaging volume dimensions, which are 775 x 775 x 300 µm.

We first evaluated sensory innervation of the prostate using conventional immunofluorescence labeling on 2D tissue sections of the mouse prostate. The markers used included PanCK to identify prostate epithelial cells, PGP9.5 to identify nerve fibers, and Hoechst or DAPI to identify nuclei (Supplementary Figures 2A,B). The 2D images illustrated the presence of nerve fibers around the prostatic acini but presented them as discontinuous short segments or dots. This fragmented representation limited our ability to estimate the architecture of the neuronal networks and the density of innervation. To address these limitations, we used an ethyl cinnamate-based optical tissue clearing workflow combined with immunofluorescence labeling (Figure 2A). Following microdissection and processing, prostate lobes were fully immersed in ethyl cinnamate for refractive index matching (Figure 2B). Three-dimensional immunofluorescence labeling for beta-3-tubulin (TUBB3), a neuronally enriched form of tubulin, revealed an extensive neuronal network innervating the ventral prostate lobe, with nerve tracts traversing through the stroma and smaller fibers contoured around epithelial acini, as illustrated clearly in the orthogonal views of the 3D image (Figure 2C and Supplementary Video 1). This improved methodology provided a comprehensive view of high-resolution, contiguous, innervation in the prostate, overcoming the limitations of conventional 2D immunofluorescence.

### 3D microarchitecture of prostate acini

**Figure 3:**
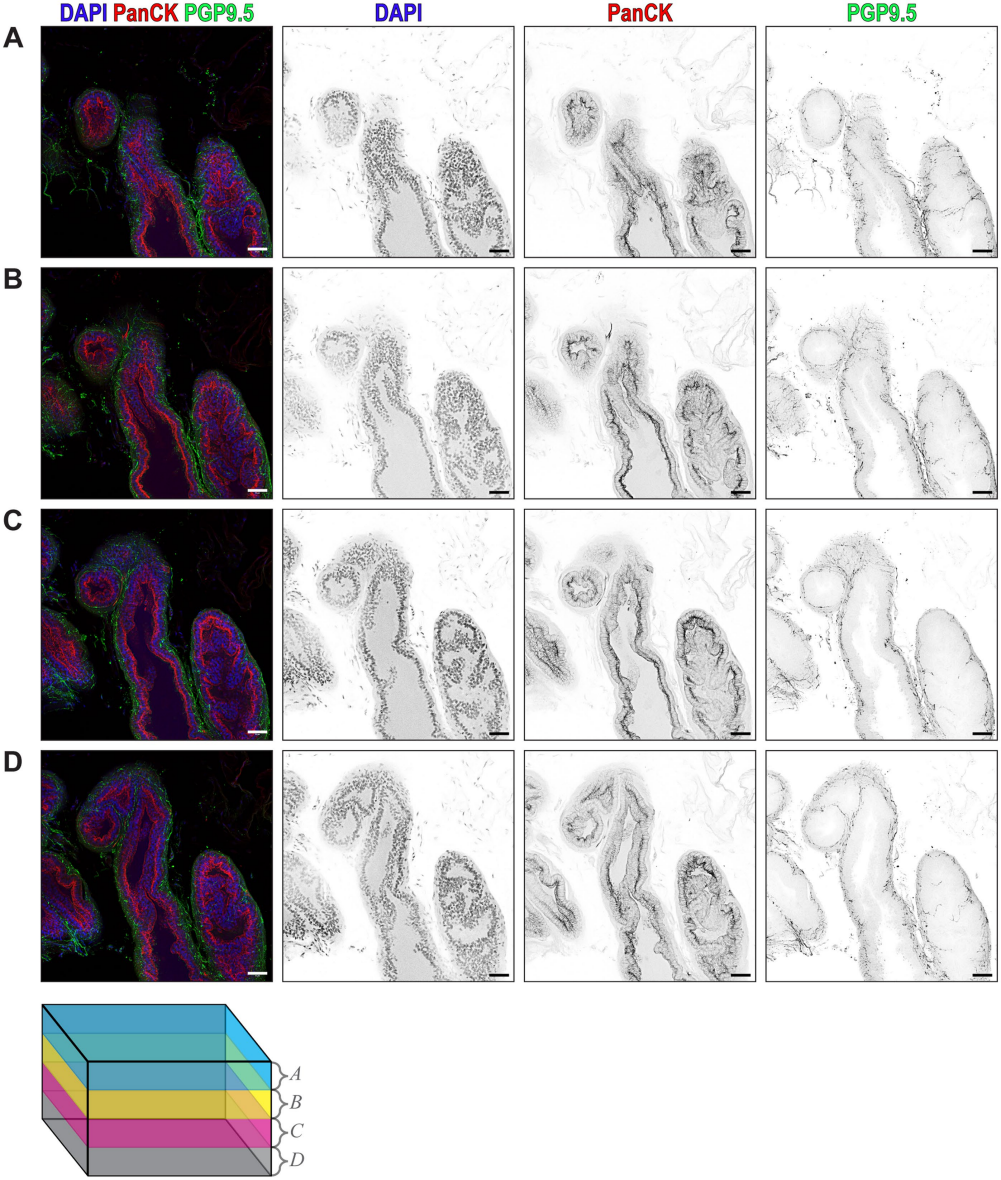
Ethyl Cinnamate-Based Optical Tissue Clearing Combined with Confocal Immunofluorescence Microscopy Reveals the 3D Microarchitecture of Prostate Acini. **(A–D)** Composite maximum intensity z-projections of four consecutive 20 µm z-stacks, with DAPI (blue) labeling nuclei, PanCK (red) labeling prostate epithelium, and PGP9.5 (green) labeling all nerves. Individual channels rendered in inverted grayscale showing DAPI, PanCK, and PGP9.5 from each 20 µm z-stack. Each image is a maximum intensity z-projection isolated from the 80 µm total imaging volume. Color-coded cube represents the z-depth of the volume corresponding to **A–D**. Scalebars represent 50 µm.

To understand the spatial relationship between prostatic nerves and the prostate epithelium, we used ethyl cinnamate-based optical tissue clearing combined with immunofluorescent confocal microscopy and labeled the mouse dorsal prostate lobe with DAPI for nuclei, PanCK for the prostatic epithelium, and PGP9.5 for peripheral nerve fibers. Three-dimensional renderings of the stained tissue reveal the detailed microarchitecture of the prostate acini and their associated nerve fibers (Supplementary Video 2). Maximum intensity projections of four 20 μm z-stacks across a total thickness of 80 μm, show nerve fibers in close proximity to the epithelium, with the densest areas of innervation near the epithelium (Figures 3A–D).

### Peptidergic sensory innervation of the mouse prostate

**Figure 4:**
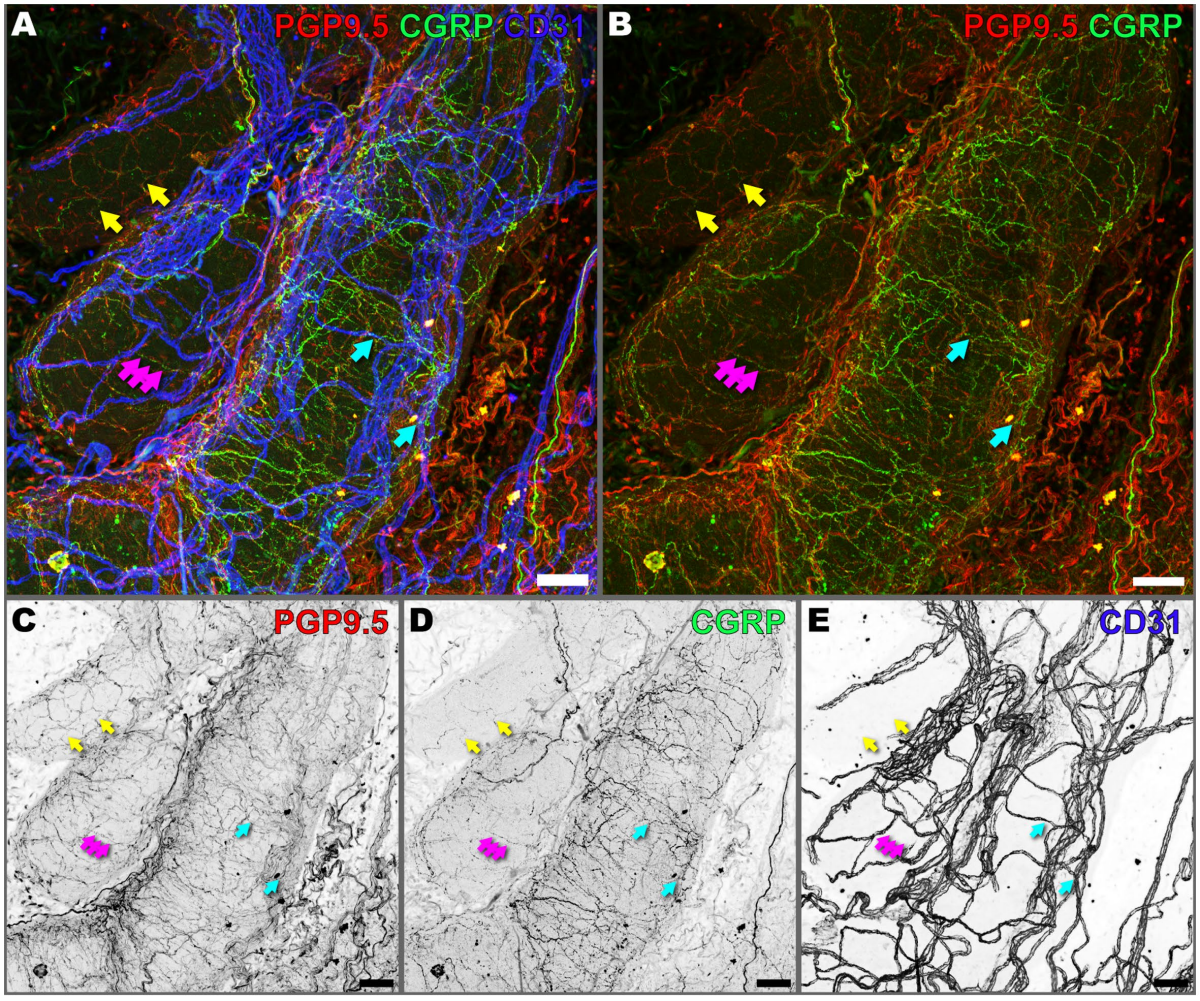
Peptidergic sensory nerves extensively innervate the mouse prostate. **(A)** Composite, and **(B)** PGP9.5 and CGRP, overlay maximum intensity z-projection images of a 300 μm deep image volume captured from cleared prostate tissue using 3D confocal microscopy, showing innervation and vasculature. PGP9.5 (red) labels all peripheral nerves, CGRP (green) labels peptidergic sensory nerves, and CD31 (blue) labels vasculature. **(C–E)** Maximum intensity z-projections of individual channels in inverted grayscale for **(C)** PGP9.5, **(D)** CGRP, and **(E)** CD31. Yellow arrows denote colocalization of CGRP and PGP9.5 in sensory nerve fibers. Cyan arrows denote close association of peptidergic sensory nerves with CD31. Magenta arrows denote arrows denote punctate CGRP signal. Images are representative of three biological replicates, *N* = 3. Scalebars represent 50 μm.

Given the important role of anterograde neuropeptide release in modulating vascular tone and permeability, we sought to investigate the spatial organization of peptidergic sensory nerves in the prostate with respect to the vasculature. In our study, we observed colocalization of CGRP in a subset of PGP9.5^+^ nerve fibers (Figure 4, yellow arrows), which confirms that peptidergic sensory nerve fibers are a subset of the total population of peripheral nerves (PGP9.5) that innervate the prostate (Figures 4A,B). However, we also observed instances where CGRP-positive signals did not appear to overlap with PGP9.5, suggesting that CGRP may be present in structures not labeled by PGP9.5. Alternatively, while expression of PGP9.5 is robust in nerve bundles, there may be less expression of the marker in fine nerve fibers which are well labeled by CGRP.

To address this discrepancy, we utilized the CalcaWT/GFP mouse model, which expresses GFP under the control of the Calca promoter, to verify the localization of CGRP-expressing neurons (Supplementary Figure 3). We observed colocalization of CGRP and GFP in peptidergic nerve fibers traversing the prostate stroma (Supplementary Figures 3A–C, magenta arrows), confirming that CGRP+ fibers correspond to peptidergic sensory nerves expressing Calca-driven GFP. Additionally, we observed peptidergic nerve fibers directly superficial to the prostatic acini with low-intensity CGRP signal (Supplementary Figures 3C,D, cyan arrows), indicating variability in CGRP expression or detection within these nerve fibers. Furthermore, we observed CGRP+ nerve fibers where GFP signal was absent in superficial sections (Supplementary Figure 3D, yellow arrows) but present in deeper sections (Supplementary Figure 3E, yellow arrows). In some instances, nerve fibers exhibited GFP signal but appeared to lack CGRP signal (Supplementary Figure 3D, blue arrows); however, closer examination revealed faint, discrete puncta of CGRP that colocalized with GFP, indicating that low levels of CGRP expression or technical factors may affect detectability. Similarly, we observed punctate CGRP signal indicative of the localization of CGRP to large dense-core vesicles being trafficked along the axons of peptidergic sensory neurons (Figure 4, magenta arrows). These peptidergic sensory nerves align in bundles that are parallel to blood vessels (CD31) (Figure 4E, cyan arrows) but are also independent of blood vessels, forming complex networks surrounding the prostatic acini (Supplementary Video 3).

**Figure 5:**
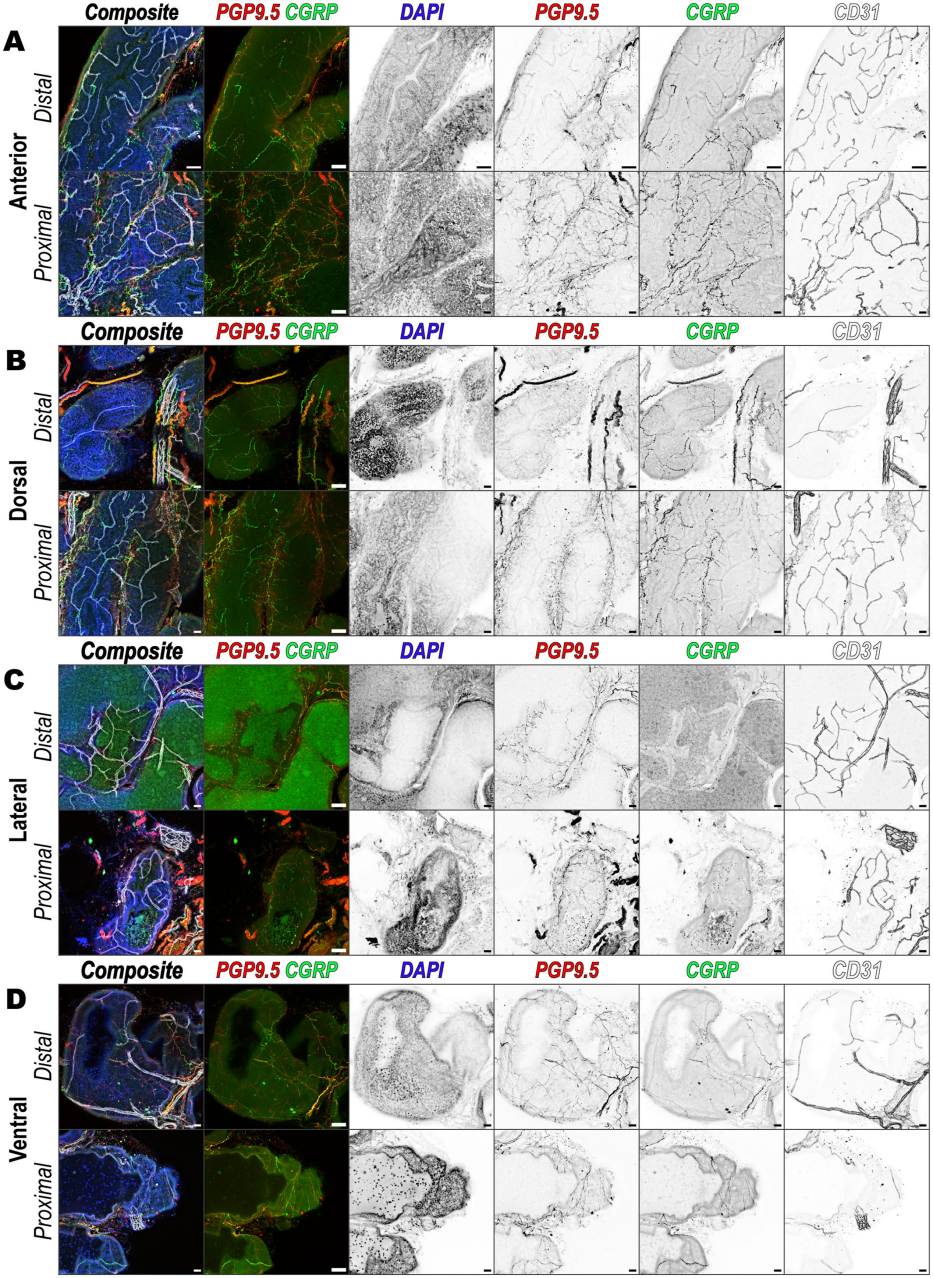
Peptidergic sensory innervation of prostatic lobes. Immunofluorescence images showing innervation patterns of the **(A)** anterior, **(B)** dorsal, **(C)** lateral, and **(D)** ventral prostate lobes from distal and proximal urethral regions, respectively. Each panel set consists of a composite overlay of all channels and an overlay of PGP9.5 and CGRP and is followed by individual channels presented in inverted grayscale. The channels are arranged left to right as follows: DAPI (nuclei, blue), PGP9.5 (nerve marker, red), CGRP (peptidergic sensory nerves, green), and CD31 (vasculature, white/gray). Each image is a maximum intensity z-projection of a 50 μm tissue volume and is representative of three biological replicates, *N* = 3, for each lobe and region. Scalebars represent 50 μm.

To identify consistency of peptidergic sensory nerve localization across the various prostate lobes and distance from the urethra, we performed 3D immunofluorescence imaging on individually microdissected prostate lobes. Focusing on proximal-urethral and distal-urethral regions, the images revealed extensive innervation by peptidergic sensory nerves with varying densities across different regions. In the anterior (Figure 5A), dorsal (Figure 5B), lateral (Figure 5C), and ventral (Figure 5D) lobe, nerve fibers labeled with PGP9.5 and CGRP were observed both nearby and distant from vascular structures labeled by CD31. This indicates that peptidergic sensory nerves are present across all prostate lobes and regions. As observed previously (Figure 4), some sensory nerve fibers are interwoven with the vasculature, while others travel independently. Although variations in CGRP signal intensity and overlap with PGP9.5 were noted among different lobes, the overall pattern indicates a widespread distribution of peptidergic sensory nerves throughout the prostate.

### Ablation of peptidergic sensory nerves

**Figure 6:**
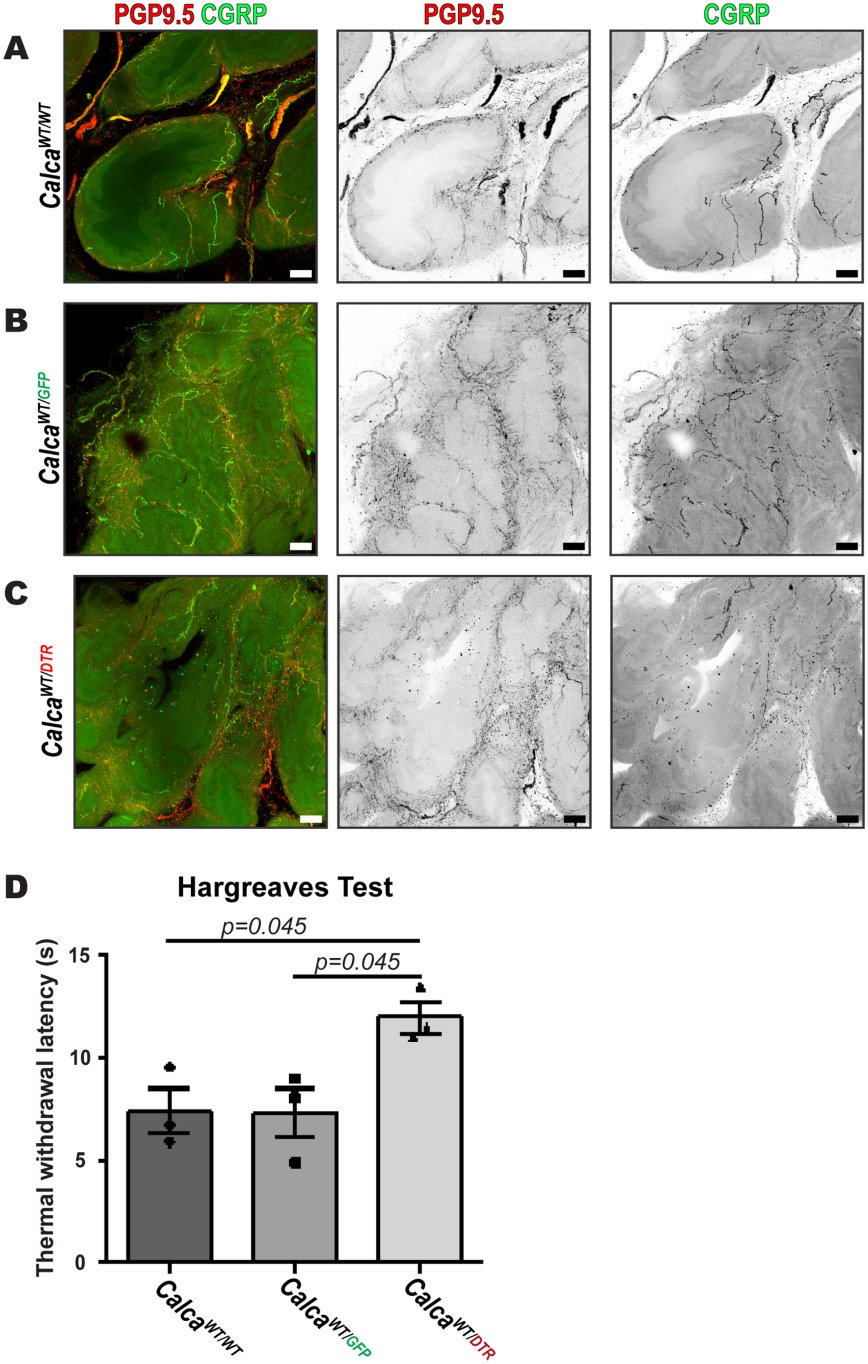
Administration of diphtheria toxin induces peptidergic sensory nerve ablation in the prostate of CalcaWT/DTR mice. **(A–C)** Maximum intensity z-projections of 20 μm acquired by fluorescence confocal microscopy of cleared mouse prostate lobes from **(A)** Calca^WT/WT^ **(B)** Calca^WT/GFP^, **(C)** and Calca^WT/DTR^ ablated mice showing PGP9.5 for all peripheral nerve fibers and CGRP for peptidergic sensory nerve fibers, and is representative of three biological replicates, *N* = 3, for each group. Scalebars represent 50 μm. **(D)** The Hargreaves test showed increased latency to paw withdrawal in response to thermal stimuli in ablated (Calca^WT/DTR^) animals compared to genotype control (Calca^WT/GFP^) and Calca^WT/WT^ control animals 21 days after diphtheria toxin injection (*n* = 3; *p < 0.05, Tukey HSD).

To understand the role of peptidergic sensory nerves in the prostate, we selectively ablated PSNs by administering DTX to transgenic mice expressing the hDTR on PSNs (Calca^WT/DTR^). Additionally, DTX was also administered to wildtype (Calca^WT/WT^) and genotype control (Calca^WT/GFP^) animals to control for any potential off target effects. The efficacy of sensory nerve ablation was assessed by deficiencies in behavioral responses to thermal stimuli and by immunofluorescence imaging of prostate tissue. 3D immunofluorescent images showed the absence of CGRP immunoreactivity in nerve fibers labeled by PGP9.5 in ablated animals (Calca^WT/DTR^) compared to intact animals (Calca^WT/WT^, Calca^WT/GFP^) (Figures 6A–C). To demonstrate that our ablation protocol resulted in loss of peptidergic sensory fibers systemically, we evaluated the responses of the mice to a thermal stimulus. The Hargreaves test confirmed sensory ablation by showing increased latency to paw withdrawal in response to thermal stimuli in ablated animals to 12.0 ± 0.8 s (Calca^WT/DTR^) compared to genotype control (Calca^WT/GFP^) and wildtype (Calca^WT/WT^) control animals at 7.3 ± 1.2 and 7.4 ± 1.1 s, respectively, 14 days the second DTX injection (Figure 6D).

### Effects of Calca haploinsufficiency and ablation of peptidergic sensory nerves on prostate histology

**Figure 7:**
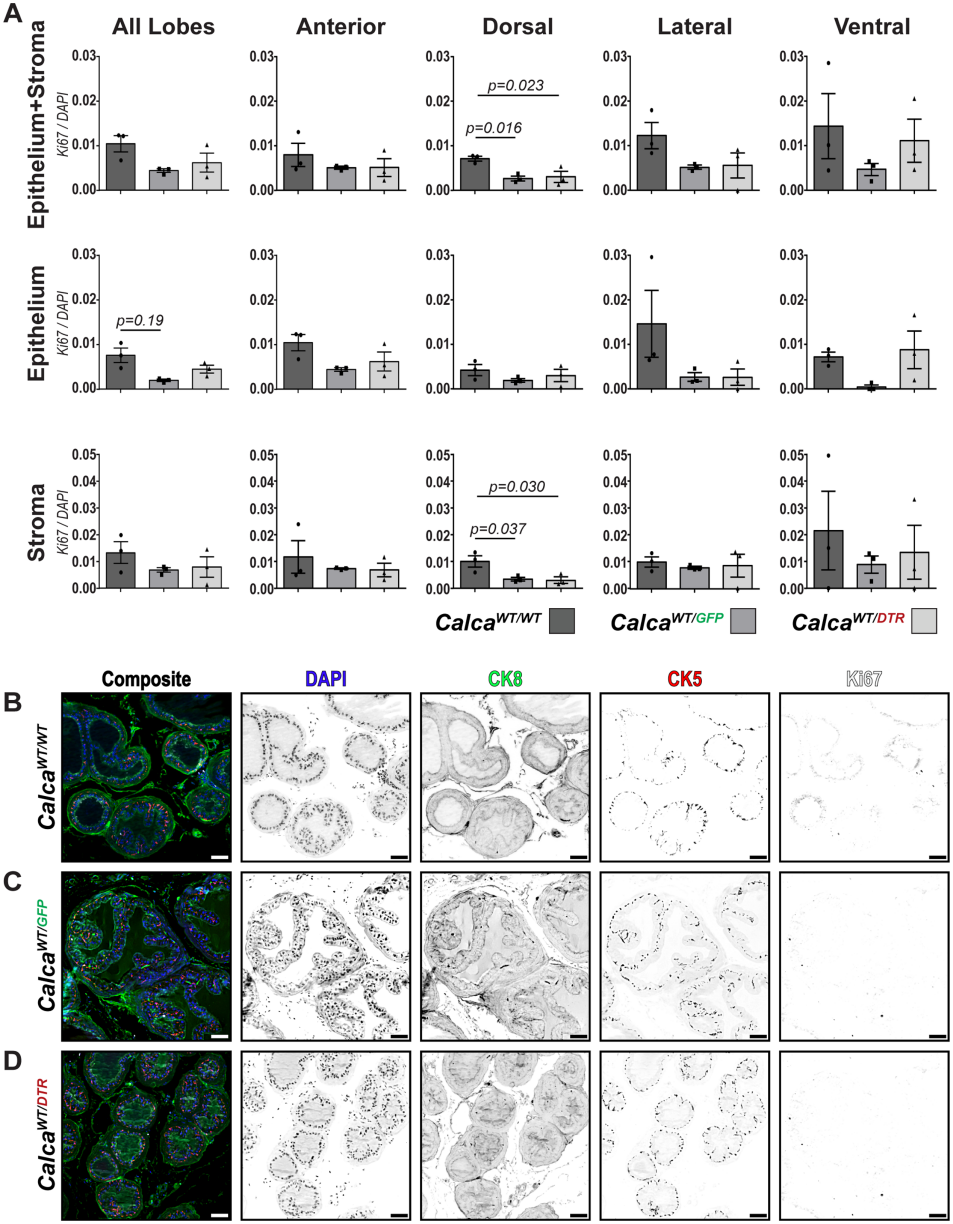
Effect of peptidergic sensory ablation on the mouse prostate. **(A)** Quantification of Ki67+ cells in different lobes (anterior, dorsal, lateral, ventral) and tissue classifications (epithelium, stroma) across WT, Calca^WT/GFP^, and Calca^WT/DTR^ groups. Statistical analysis was performed using one-way ANOVA with Dunnett’s multiple comparisons test. Each point denotes represebts a single biological replicate. **(B–D)** Representative images (*N* = 3) showing DAPI (blue) for nuclei, CK8 (green) for luminal cells, CK5 (red) for basal cells, and Ki67 (white) for proliferating cells and inverted grayscale images of individual channels for the dorsal lobe in **(B)** wild-type (Calca^WT/WT^), **(C)** Calca^WT/GFP^ (CGRP haploinsufficient), and **(D)** Calca^WT/DTR^ (sensory-nerve-ablated) animals. Scalebars represent 50 μm.

Once we established that the ablation model was successful, we proceeded to examine the effects of sensory ablation on prostate proliferation. As mentioned previously, our model for ablation of sensory neurons depends upon a transgene insertion on one of the two Calca alleles. While previous reports using this model did not observe obvious changes in nociceptive behavior with this transgene (McCoy et al., 2012), inclusion of Calca^WT/GFP^ mice in our study was critical to control for a potential developmental deficiency in CGRP signaling. We sectioned half of the prostate and evaluated 2–3 sections for the presence of Ki67 to determine whether Calca haploinsufficiency or sensory nerve ablation alters cell proliferation in either the epithelial or stromal compartments within each of the prostatic lobes. Given the potentially pleiotropic effect of sensory nerve-derived neuropeptides, we performed whole slide imaging and utilized Cellpose, an AI-based deep learning model, for cell segmentation, along with QuPath for object and pixel classification (Stringer et al., 2021; Bankhead et al., 2017). This allowed us to conduct a more comprehensive evaluation of prostate tissue while ensuring that we had the sensitivity to detect potentially low frequency variations in Ki67 positivity. Representative images were collected from each treatment group. The epithelial compartment was defined as that including expression of the cytokeratin 5 and/or 8 (Figures 7B–D). We counted the number of Ki67+ cells and found that Calca haploinsufficiency diminished the number of proliferative cells when examining the dorsal lobe as a whole. When we further examined the localization of this effect, we observed a significant decrease in the stromal compartment, but only a trend within the epithelial compartment. Additionally, Calca haploinsufficiency only elicited a trend of diminished Ki67^+^ cells in the lateral and ventral epithelium. Diminution of sensory nerves via ablation did not appear to have an effect on Ki67+ cell numbers above the effects of Calca haploinsufficiency in any of the lobes (Figure 7A).

## Discussion

In the current study, we found extensive sensory innervation in all lobes of the mouse prostate. To determine the functional role of these sensory neurons, we used an intersectional genetic approach to ablate the nerves and then assessed changes in the prostate by measuring differences in cellular proliferation. Surprisingly, we observed a decrease in epithelial-specific proliferation in the genotype control compared to wildtype mice, but no additional effects of sensory ablation beyond the effects of *Calca* allelic replacement were observed. The genotype control was constructed such that one allele of *Calca* was replaced by a non-coding transgene, suggesting a possible role for CGRP in prostate homeostasis.

Prostate disease will affect a majority of the male population in their lifetime. Benign prostatic hyperplasia (BPH) affects 50% of males by age 50, while 13% of males will be diagnosed with prostate cancer at some point in their lives (McVary, 2006; National Cancer Institute, 2024). In addition, chronic pelvic pain syndrome (CPPS) continues to be a persistent health concern. While treatment options are available, there is still a substantial refractory population for each prostate disorder, emphasizing the need for further understanding of the biology and tissue microenvironment of the prostate (Pound et al., 1999; Boorjian et al., 2011; Ginzburg et al., 2012; Liesenfeld et al., 2017; Penning, 2015). Researchers have studied the tissue microenvironment of the prostate for years to understand how proliferative prostatic diseases begin and progress, and how the microenvironment may impact resistance to therapy (Corn, 2012; Yadav et al., 2018; Hofman and Emmett, 2019). However, studies of the prostate microenvironment have generally neglected a potential role for sensory nerves in prostatic disease (Erin et al., 2004; Zhao et al., 2014; Keskinov et al., 2016; Saloman et al., 2016; Prazeres et al., 2020; Ayala et al., 2008; Zahalka et al., 2017; Zahalka et al., 2020; Magnon et al., 2013).

In light of this gap and the previously reported sparsity of prostatic sensory innervation in the literature (Blasko et al., 2023; Pennefather et al., 2000; Wegner et al., 2017; McVary et al., 1998), our findings were rather unexpected. Perhaps they should not have been, as it is known that neurovascular bundles, which include sensory nerves, track with glandular growth during prostatic development (Turco et al., 2019), supporting the notion that sensory nerves may be present but underappreciated. Of note, our work is the first we are aware of that has used the tissue clearing 3D methodology to visualize sensory nerves in prostate, and this may be part of the reason that they were underidentified previously. Our current findings demonstrate extensive peptidergic sensory innervation within the prostate of 11- to 12-month-old mice, revealed through tissue clearing of individual prostate lobes. In addition to being present in and around the pelvic ganglia and stromal compartment (Wegner et al., 2017; Bertrand et al., 2020), we found peptidergic sensory nerves immediately juxtaposed to and surrounding prostatic acini. Notably, peptidergic sensory innervation appears to be denser in the dorsal prostate compared to other lobes, but ongoing quantitative studies are necessary to validate this observation. As expected, sensory nerves in the stroma were limited to specific innervation of vasculature as indicated by adjacent signal from PGP9.5/CGRP and CD31 labeling. We also observed instances where sensory innervation extended into the smooth muscle layer around each acinus. Taken together, the positioning we found could allow sensory neurons to detect changes in muscle tension and contraction, which could be important for regulating the secretion of prostatic fluid and maintaining the structural integrity of the acini.

To ablate peptidergic sensory neurons in an adult mouse, we adopted a DTX model for ablation. In short, a Calca-fl-eGFP-STOP-fl-DTR mouse is crossed with an Advillin-Cre mouse. Advillin-driven cre-recombinase excises a STOP codon to facilitate DTR expression (Hunter et al., 2018). Advillin is expressed in all neural crest derived cells (Coarfa et al., 2018); however, the power of this mouse design is that expression of the DTR is limited to cell types that express both CGRP and advillin, which limits DTR expression to peptidergic sensory neurons. As mice do not naturally express hDTR, DTX administration selectively ablates CGRP+ neurons, and this effect is shown by a marked reduction in sensory innervation in the prostate and by deficiencies in thermal response to noxious heat. Upon deciding to use this DTX model for sensory ablation, we understood that one allele for *Calca* was replaced with a transgene that introduced the hDTR. Previous reports did not detect a change in nociceptive behaviors in the Calca^WT/GFP^ mice (Coarfa et al., 2018), and we confirmed that there was no difference in thermal withdrawal latencies between wildtype and Calca^WT/GFP^ mice in our study, therefore there was little evidence to suggest that allelic replacement affected the availability of CGRP.

The most profound effects that we observed in our study were the effects of the *Calca* allelic replacement on the number of proliferative prostatic cells, suggesting that the impact on basal proliferation may be largely due to CGRP decreases. Full sensory nerve ablation had very little additional effect beyond the haploinsufficiency CGRP. These findings suggest a potential homeostatic role of the release of CGRP from peptidergic sensory nerves within the prostate. Again, we did not observe a difference between the Calca haploinsufficient and ablated mice, confirming that changes in epithelial proliferation in Calca^WT/DTR^ mice are not a consequence of excitotoxicity or tissue inflammatory response associated with sensory neuron death—a concern in other models of sensory ablation. Clearly, even when we observed effects of sensory neuron dysregulation on prostatic proliferation, the effect sizes were small. We anticipate that we will observe larger effects of sensory neuron manipulation in the presence of proinflammatory stimuli such as aging, T-cell activation, cancer, or infection. Regardless, the effect of CGRP loss on epithelial proliferation likely impacts overall epithelial biology, and future work will determine the specific mechanisms by which CGRP functions.

Previous attempts to either denervate or ablate neurons within the prostate largely focused on autonomic nerves (Magnon et al., 2013). In rats, chemical denervation via Botox treatment or surgical denervation via excision of the major pelvic ganglia, resulted in glandular atrophy, alterations in protein secretion, and ultrastructural changes in the epithelium (Gamse, 1982; Nagy and van der Kooy, 1983; Szallasi and Blumberg, 1999). In mice, chemical denervation of sympathetic nerves via systemic administration of 6-hydroxydopamine or surgical transection of the hypogastric nerves resulted in delayed proliferation of prostate cancer xenografts, whereas parasympathetic activation via systemic administration of carbachol results in the increased proliferation of xenografts in the prostate and increased dissemination of cancer cells into the lymph nodes (Sanchez et al., 2005). While several of these models of autonomic denervation likely affect sensory neuron innervation of the prostate, studies to determine the specific contributions of sensory neurons within the prostate had not been completed. Systemic administration of capsaicin or resiniferatoxin to adult or neonatal mice has been used to ablate sensory nerves (Gamse, 1982, Nagy and van der Kooy, 1983, Szallasi and Blumberg, 1999); however, this approach can have off target effects for non-neuronal cells that express the ion channel activated by capsaicin, the TRPV1 (McCoy et al., 2012). All of these limitations with surgical and excitotoxic ablation support our rationale for using the Calca^WT/DTR^ model to ablate peptidergic sensory nerves.

Sensory neuron activity within the prostate could be crucial for coordinating the response of the prostate to various physiological stimuli, altering growth and differentiation (Shariff and Ather, 2006). Sensory neurons undergo tonic release of low levels of neurotransmitters into the microenvironment that they innervate (Holzer and Maggi, 1998), and when stimulated, anterogradely release bolus amounts of these neurotransmitters. There are several neurotransmitters that are commonly released from sensory neurons, including SP, CGRP, and glutamate (Hua, 1986; Fernandez-Montoya et al., 2017). In addition to neurotransmitters, sensory neurons have also been documented to release cytokines and chemokines, including IL-1β (Copray et al., 2001), IL-6 (Hu et al., 2020), CCL2 (Zhang et al., 2019), and CXCL1 (Fukuda et al., 2013; Sample et al., 2014). In tissues such as bone, neurotransmitter release is essential for functions such as load-dependent bone remodeling (Fukuda et al., 2013; Sample et al., 2014), even in the absence of pain, which is commonly perceived as the primary function of sensory neurons. The genotype control for sensory ablation suggests that altered CGRP signaling can affect the prostate, but it is unclear how changes in CGRP might alter other neurotransmitters or cytokines and chemokines in proximity to the nerves. Furthermore, there could be compensatory changes in non-peptidergic sensory or autonomic nerves with developmental decreases in CGRP expression (Cortelli et al., 2013). CGRP typically activates Gsα signaling upon activation of the calcitonin receptor-like and receptor activity-modifying protein 1 receptor complex. This activation induces a pronounced vasodilation, which increases blood flow and contributes to tissue perfusion (Haass and Skofitsch, 1985). Interestingly, CGRP also modulates immune cell function by inhibiting the release of pro-inflammatory cytokines from macrophages and other immune cells (Li et al., 2020; Veltri et al., 2005; Xu et al., 2024), thus potentially exerting anti-inflammatory effects. CGRP also has an anti-fibrotic role in multiple tissues (Li et al., 2020; Veltri et al., 2005; Xu et al., 2024). Collectively, the actions of CGRP help coordinate the body’s response to injury but can modulate chronic inflammation and tissue remodeling with prolonged or excessive signaling. While CGRP signaling has been proposed to promote prostate cancer progression and metastasis (Logan et al., 2013; Zhu et al., 2021), there is limited information about its other roles in the prostate, which remains an area for future investigation.

### Future directions

Overall, our findings suggest that sensory neuron activity in the prostate may be critical for regulating tissue homeostasis and responding to various stimuli. These results have significant implications for understanding prostate diseases like BPH, CPPS, and prostate cancer, where sensory nerves may play a role in disease progression and therapeutic resistance. While our present study provides new insights into sensory nerve function in the prostate, our future research will focus on further characterizing these mechanisms in disease models, including aging, inflammation, and cancer.

## Data Availability

The raw data supporting the conclusions of this article will be made available by the authors, without undue reservation.

## References

[ref1] Ayala G. E. Dai H. Powell M. Li R. Ding Y. Wheeler T. M.. (2008). Cancer-related axonogenesis and neurogenesis in prostate cancer. Clin. Cancer Res. 14, 7593–7603. doi: 10.1158/1078-0432.CCR-08-1164 19047084

[ref2] Bahmad H. F. Jalloul M. Azar J. Moubarak M. M. Samad T. A. Mukherji D.. (2021). Tumor microenvironment in prostate cancer: toward identification of novel molecular biomarkers for diagnosis, prognosis and therapy development. Front. Genet. 12:652747. doi: 10.3389/fgene.2021.652747 33841508 PMC8033163

[ref3] Bankhead P. Loughrey M. B. Fernández J. A. Dombrowski Y. Mcart D. G. Dunne P. D.. (2017). Qupath: open source software for digital pathology image analysis. Sci. Rep. 7:16878. doi: 10.1038/s41598-017-17204-5, PMID: 29203879 PMC5715110

[ref4] Bertrand M. M. Korajkic N. Osborne P. B. Keast J. R. (2020). Functional segregation within the pelvic nerve of male rats: a Meso- and microscopic analysis. J. Anat. 237, 757–773. doi: 10.1111/joa.13221, PMID: 32598494 PMC7495281

[ref5] Blasko F. Krivosikova L. Babal P. Breza J. Trebaticky B. Kuruc R.. (2023). Innervation density and types of nerves in prostate cancer. Neoplasma 70, 787–795. doi: 10.4149/neo_2023_231116N593, PMID: 38247335

[ref6] Boorjian S. A. Thompson R. H. Tollefson M. K. Rangel L. J. Bergstralh E. J. Blute M. L.. (2011). Long-term risk of clinical progression after biochemical recurrence following radical prostatectomy: the impact of time from surgery to recurrence. Eur. Urol. 59, 893–899. doi: 10.1016/j.eururo.2011.02.026, PMID: 21388736

[ref7] Brain S. D. (1997). Sensory neuropeptides: their role in inflammation and wound healing. Immunopharmacology 37, 133–152. doi: 10.1016/S0162-3109(97)00055-6, PMID: 9403332

[ref8] Bray F. Laversanne M. Sung H. Ferlay J. Siegel R. L. Soerjomataram I.. (2024). Global cancer statistics 2022: globocan estimates of incidence and mortality worldwide for 36 cancers in 185 countries. CA Cancer J. Clin. 74, 229–263. doi: 10.3322/caac.21834, PMID: 38572751

[ref9] Burri O. Sobolewski P. Fehlmann T. (2024). Biop/qupath-extension-cellpose: improved label image reading and code linting (V0.9.6). Zenodo. doi: 10.5281/Zenodo.13752036

[ref10] Chiu I. M. Von Hehn C. A. Woolf C. J. (2012). Neurogenic inflammation and the peripheral nervous system in host defense and immunopathology. Nat. Neurosci. 15, 1063–1067. doi: 10.1038/nn.3144, PMID: 22837035 PMC3520068

[ref11] Coarfa C. Florentin D. Putluri N. Ding Y. Au J. He D.. (2018). Influence of the neural microenvironment on prostate Cancer. Prostate 78, 128–139. doi: 10.1002/pros.23454, PMID: 29131367 PMC5836952

[ref12] Copray J. C. Mantingh I. Brouwer N. Biber K. Küst B. M. Liem R. S.. (2001). Expression of Interleukin-1 Beta in rat dorsal root ganglia. J. Neuroimmunol. 118, 203–211. doi: 10.1016/S0165-5728(01)00324-1 11498255

[ref13] Corn P. G. (2012). The tumor microenvironment in prostate Cancer: elucidating molecular pathways for therapy development. Cancer Manag. Res. 4, 183–193. doi: 10.2147/CMAR.S32839, PMID: 22904640 PMC3421469

[ref14] Cortelli P. Giannini G. Favoni V. Cevoli S. Pierangeli G. (2013). Nociception and autonomic nervous system. Neurol. Sci. 34, 41–46. doi: 10.1007/s10072-013-1391-z 23695044

[ref15] Cunha G. R. (1972). Epithelio-mesenchymal interactions in primordial gland structures which become responsive to androgenic stimulation. Anat. Rec. 172, 179–195. doi: 10.1002/ar.1091720206 5012433

[ref16] Cunha G. R. Lung B. Reese B. (1980). Glandular epithelial induction by embryonic mesenchyme in adult bladder epithelium of Balb/C mice. Investig. Urol. 17, 302–304, PMID: 6153176

[ref17] Cunha G. R. Vezina C. M. Isaacson D. Ricke W. A. Timms B. G. Cao M.. (2018). Development of the human prostate. Differentiation 103, 24–45. doi: 10.1016/j.diff.2018.08.005, PMID: 30224091 PMC6234090

[ref18] Duan J.-X. Zhou Y. Zhou A.-Y. Guan X.-X. Liu T. Yang H.-H.. (2017). Calcitonin gene-related peptide exerts anti-inflammatory property through regulating murine macrophages polarization in vitro. Mol. Immunol. 91, 105–113. doi: 10.1016/j.molimm.2017.08.020 28892747

[ref19] Erin N. Boyer P. J. Bonneau R. H. Clawson G. A. Welch D. R. (2004). Capsaicin-mediated denervation of sensory neurons promotes mammary tumor metastasis to Lung and heart. Anticancer Res. 24, 1003–1009, PMID: 15161056

[ref20] Fernandez-Montoya J. Avendano C. Negredo P. (2017). The glutamatergic system in primary somatosensory neurons and its involvement in sensory input-dependent plasticity. Int. J. Mol. Sci. 19:69. doi: 10.3390/ijms19010069, PMID: 29280965 PMC5796019

[ref21] Fibbi B. Penna G. Morelli A. Adorini L. Maggi M. (2010). Chronic inflammation in the pathogenesis of benign prostatic hyperplasia. Int. J. Androl. 33, 475–488. doi: 10.1111/j.1365-2605.2009.00972.x 19508330

[ref22] Fukuda T. Takeda S. Xu R. Ochi H. Sunamura S. Sato T.. (2013). Sema3a regulates bone-mass accrual through sensory innervations. Nature 497, 490–493. doi: 10.1038/nature12115 23644455

[ref23] Gal P. Varinska L. Faber L. Novak S. Szabo P. Mitrengova P.. (2017). How signaling molecules regulate tumor microenvironment: parallels to wound repair. Molecules 22:11-1818. doi: 10.3390/molecules22111818, PMID: 29072623 PMC6150347

[ref24] Gamse R. (1982). Capsaicin and nociception in the rat and mouse. Possible role of substance P. Naunyn Schmiedeberg's Arch. Pharmacol. 320, 205–216. doi: 10.1007/BF00510129 6182473

[ref25] Ge R. Wang Z. Cheng L. (2022). Tumor microenvironment heterogeneity an important mediator of prostate Cancer progression and therapeutic resistance. Npj Precision Oncology 6:31. doi: 10.1038/s41698-022-00272-w, PMID: 35508696 PMC9068628

[ref26] Ginzburg S. Nevers T. Staff, I Tortora J. Champagne A. Kesler S. S.. (2012). Prostate Cancer biochemical recurrence rates after robotic-assisted laparoscopic radical prostatectomy. Jsls 16, 443–450. doi: 10.4293/108680812X13462882736538, PMID: 23318071 PMC3535788

[ref27] Grishina I. B. Kim S. Y. Ferrara C. Makarenkova H. P. Walden P. D. (2005). Bmp7 inhibits branching morphogenesis in the prostate gland and interferes with notch signaling. Dev. Biol. 288, 334–347. doi: 10.1016/j.ydbio.2005.08.018, PMID: 16324690 PMC2644052

[ref28] Haase R. Royer L. A. Steinbach P. Schmidt D. Dibrov A. Schmidt U.. (2020). Clij: Gpu-accelerated image processing for everyone. Nat. Methods 17, 5–6. doi: 10.1038/s41592-019-0650-1, PMID: 31740823

[ref29] Haass M. Skofitsch G. (1985). Cardiovascular effects of calcitonin gene-related peptide in the pithed rat: comparison with substance P. Life Sci. 37, 2085–2090. doi: 10.1016/0024-3205(85)90580-6, PMID: 2415796

[ref30] Hallberg D. Pernow B. (1975). Effect of substance P on various vascular beds in the dog. Acta Physiol. Scand. 93, 277–285. doi: 10.1111/j.1748-1716.1975.tb05816.x, PMID: 1146575

[ref31] Hofman M. S. Emmett L. (2019). Tumour heterogeneity and resistance to therapy in prostate Cancer: a fundamental limitation of prostate-specific membrane antigen Theranostics or a key strength? Eur. Urol. 76, 479–481. doi: 10.1016/j.eururo.2019.07.030 31351666

[ref32] Holzer P. Maggi C. A. (1998). Dissociation of dorsal root ganglion neurons into afferent and efferent-like neurons. Neuroscience 86, 389–398, PMID: 9881854 10.1016/s0306-4522(98)00047-5

[ref33] Hu H. Cui Y. Yang J. Cao Y. (2022). Loss of the sympathetic signal produces sterile inflammation of the prostate. Front. Mol. Neurosci. 15:855376. doi: 10.3389/fnmol.2022.855376, PMID: 35620446 PMC9127543

[ref34] Hu Z. Deng N. Liu K. Zhou N. Sun Y. Zeng W. (2020). Cntf-Stat3-Il-6 Axis mediates Neuroinflammatory Cascade across Schwann cell-neuron-microglia. Cell Rep. 31:107657. doi: 10.1016/j.celrep.2020.107657, PMID: 32433966

[ref35] Hua X. Y. (1986). Tachykinins and calcitonin gene-related peptide in relation to peripheral functions of capsaicin-sensitive sensory neurons. Acta Physiol. Scand. Suppl. 551, 1–45, PMID: 2430427

[ref36] Huang J. Brenna C. Khan A. U. M. Daniele C. Rudolf R. Heuveline V.. (2019). A cationic near infrared fluorescent agent and ethyl-Cinnamate tissue clearing protocol for vascular staining and imaging. Sci. Rep. 9:0077-0018. doi: 10.1038/s41598-018-36741-1 PMC6345820 30679514

[ref37] Hunter D. V. Smaila B. D. Lopes D. M. Takatoh J. Denk F. Ramer M. S. (2018). Advillin is expressed in all adult neural crest-derived neurons. Eneuro 5:Eneuro.0077-18. doi: 10.1523/eneuro.0077-18.2018 PMC6135988 30221190

[ref38] Ittmann M. (2018). Anatomy and histology of the human and murine prostate. Cold Spring Harb. Perspect. Med. 8:0077-0018. doi: 10.1101/cshperspect.a030346 PMC5932577 29038334

[ref39] Jen P. Y. P. Dixon J. S. Gosling J. A. (1995). Development of peptide-containing nerves in the human fetal vas deferens and seminal vesicle. Br. J. Urol. 75, 378–385. doi: 10.1111/j.1464-410X.1995.tb07353.x 7735805

[ref40] Jerde T. J. Bushman W. (2009). Il-1 induces Igf-dependent epithelial proliferation in prostate development and reactive hyperplasia. Sci. Signal. 2:Ra49. doi: 10.1126/scisignal.2000338 19724062 PMC2949294

[ref41] Keskinov A. A. Tapias V. Watkins S. C. Ma Y. Shurin M. R. Shurin G. V. (2016). Impact of the sensory neurons on melanoma growth in vivo. PLoS One 11, –e0156095. doi: 10.1371/journal.pone.0156095, PMID: 27227315 PMC4882065

[ref42] Kjartansson J. Dalsgaard C. J. Jonsson C. E. (1987). Decreased survival of experimental critical flaps in rats after sensory denervation with capsaicin. Plast. Reconstr. Surg. 79, 218–221. doi: 10.1097/00006534-198702000-00012 3543981

[ref43] Lembeck F. Holzer P. (1979). Substance P as neurogenic mediator of Antidromic vasodilation and neurogenic plasma extravasation. Naunyn Schmiedeberg's Arch. Pharmacol. 310, 175–183. doi: 10.1007/BF00500282, PMID: 93706

[ref44] Li W. Zhang Z. Li X. Cai J. Li D. Du J.. (2020). Cgrp derived from cardiac fibroblasts is an endogenous suppressor of cardiac fibrosis. Cardiovasc. Res. 116, 1335–1348. doi: 10.1093/cvr/cvz234, PMID: 31504241

[ref45] Liesenfeld L. Kron M. Gschwend J. E. Herkommer K. (2017). Prognostic factors for biochemical recurrence more than 10 years after radical prostatectomy. J. Urol. 197, 143–148. doi: 10.1016/j.juro.2016.07.004 27418452

[ref46] Logan M. Anderson P. D. Saab S. T. Hameed O. Abdulkadir S. A. (2013). Ramp1 is a direct Nkx3.1 target gene up-regulated in prostate Cancer that promotes tumorigenesis. Am. J. Pathol. 183, 951–963. doi: 10.1016/j.ajpath.2013.05.021, PMID: 23867798 PMC3763771

[ref47] Luo W. Rodriguez M. Valdez J. M. Zhu X. Tan K. Li D.. (2013). Lgr4 is a key regulator of prostate development and prostate stem cell differentiation. Stem Cells 31, 2492–2505. doi: 10.1002/stem.1484, PMID: 23897697 PMC3934101

[ref48] Magnon C. Hall S. J. Lin J. Xue X. Gerber L. Freedland S. J.. (2013). Autonomic nerve development contributes to prostate Cancer progression. Science 341:1236361. doi: 10.1126/science.1236361, PMID: 23846904

[ref49] Masselink W. Reumann D. Murawala P. Pasierbek P. Taniguchi Y. Bonnay F.. (2019). Broad applicability of a streamlined ethyl Cinnamate-based clearing procedure. Development 146:Dev166884. doi: 10.1242/dev.166884 30665888 PMC7115989

[ref50] Mccoy E. S. Taylor-Blake B. Street S. E. Pribisko A. L. Zheng J. Zylka M. J. (2013). Peptidergic Cgrpalpha primary sensory neurons encode heat and itch and Tonically suppress sensitivity to cold. Neuron 78, 138–151. doi: 10.1016/j.neuron.2013.01.030, PMID: 23523592 PMC3628403

[ref51] Mccoy E. S. Taylor-Blake B. Zylka M. J. (2012). Cgrpalpha-expressing sensory neurons respond to stimuli that evoke sensations of pain and itch. PLoS One 7:E36355. doi: 10.1371/journal.pone.0036355, PMID: 22563493 PMC3341357

[ref52] McNeal J. E. (1981). The zonal anatomy of the prostate. Prostate 2, 35–49. doi: 10.1002/pros.2990020105 7279811

[ref53] McVary K. T. (2006). Bph: epidemiology and comorbidities. Am. J. Manag. Care 12, S122–S128, PMID: 16613526

[ref54] McVary K. T. Mckenna K. E. Lee C. (1998). Prostate innervation. Prostate Suppl. 8, 2–13, PMID: 9690657

[ref55] Nagy J. I. Van Der Kooy D. (1983). Effects of neonatal capsaicin treatment on nociceptive thresholds in the rat. J. Neurosci. 3, 1145–1150. doi: 10.1523/JNEUROSCI.03-06-01145.1983, PMID: 6133918 PMC6564618

[ref56] National Cancer Institute (2024). Surveillance, E., and end results program. Cancer stat facts: prostate cancer. Available at: https://seer.cancer.gov/statfacts/html/prost.html (Accessed July 5, 2024).

[ref57] Oliveira D. S. Dzinic S. Bonfil A. I. Saliganan A. D. Sheng S. Bonfil R. D. (2016). The mouse prostate: a basic anatomical and histological guideline. Bosn. J. Basic Med. Sci. 16, 8–13. doi: 10.17305/bjbms.2016.917, PMID: 26773172 PMC4765945

[ref58] Pennefather J. N. Lau W. A. K. Mitchelson F. Ventura S. (2000). The autonomic and sensory innervation of the smooth muscle of the prostate gland: a review of pharmacological and histological studies. J. Auton. Pharmacol. 20, 193–206. doi: 10.1046/j.1365-2680.2000.00195.x, PMID: 11260358

[ref59] Penning T. M. (2015). Mechanisms of drug resistance that target the androgen axis in castration resistant prostate cancer (Crpc). J. Steroid Biochem. Mol. Biol. 153, 105–113. doi: 10.1016/j.jsbmb.2015.05.010, PMID: 26032458 PMC4568163

[ref60] Pound C. R. Partin A. W. Eisenberger M. A. Chan D. W. Pearson J. D. Walsh P. C. (1999). Natural history of progression after Psa elevation following radical prostatectomy. JAMA 281, 1591–1597. doi: 10.1001/jama.281.17.1591, PMID: 10235151

[ref61] Prazeres P. H. D. M. Leonel C. Silva W. N. Rocha B. G. S. Santos G. S. P. Costa A. C.. (2020). Ablation of sensory nerves favours melanoma progression. J. Cell. Mol. Med. 24, 9574–9589. doi: 10.1111/jcmm.15381, PMID: 32691511 PMC7520271

[ref62] Reeves F. A. Battye S. Roth H. Peters J. S. Hovens C. Costello A. J.. (2019). Prostatic nerve subtypes independently predict biochemical recurrence in prostate cancer. J. Clin. Neurosci. 63, 213–219. doi: 10.1016/j.jocn.2019.01.052 30772200

[ref63] Richardson J. D. Vasko M. R. (2002). Cellular mechanisms of neurogenic inflammation. J. Pharmacol. Exp. Ther. 302, 839–845. doi: 10.1124/jpet.102.032797 12183638

[ref64] Saloman J. L. Albers K. M. Li D. Hartman D. J. Crawford H. C. Muha E. A.. (2016). Ablation of sensory neurons in a genetic model of pancreatic ductal adenocarcinoma slows initiation and progression of cancer. Proc. Natl. Acad. Sci. 113, 3078–3083. doi: 10.1073/pnas.1512603113, PMID: 26929329 PMC4801275

[ref65] Sample S. J. Heaton C. M. Behan M. Bleedorn J. A. Racette M. A. Hao Z.. (2014). Role of calcitonin gene-related peptide in functional adaptation of the skeleton. PLoS One 9:E113959. doi: 10.1371/journal.pone.0113959, PMID: 25536054 PMC4275203

[ref66] Sanchez M. G. Sanchez A. M. Collado B. Malagarie-Cazenave S. Olea N. Carmena M. J.. (2005). Expression of the transient receptor potential vanilloid 1 (Trpv1) in Lncap and Pc-3 prostate cancer cells and in human prostate tissue. Eur. J. Pharmacol. 515, 20–27. doi: 10.1016/j.ejphar.2005.04.010, PMID: 15913603

[ref67] Schindelin J. Arganda-Carreras I. Frise E. Kaynig V. Longair M. Pietzsch T.. (2012). Fiji: an open-source platform for biological-image analysis. Nat. Methods 9, 676–682. doi: 10.1038/nmeth.2019, PMID: 22743772 PMC3855844

[ref68] Shariff A. H. Ather M. H. (2006). Neuroendocrine differentiation in prostate cancer. Urology 68, 2–8. doi: 10.1016/j.urology.2006.02.002 16844446

[ref69] Stringer C. Wang T. Michaelos M. Pachitariu M. (2021). Cellpose: a generalist algorithm for cellular segmentation. Nat. Methods 18, 100–106. doi: 10.1038/s41592-020-01018-x 33318659

[ref70] Szallasi A. Blumberg P. M. (1999). Vanilloid (capsaicin) receptors and mechanisms. Pharmacol. Rev. 51, 159–212, PMID: 10353985

[ref71] Thomson A. A. Marker P. C. (2006). “Branching morphogenesis of the prostate” in Branching morphogenesis. ed. Davies J. A. (Springer US: Boston, MA). 10.1111/j.1432-0436.2006.00101.x 16916376

[ref72] Toivanen R. Shen M. M. (2017). Prostate organogenesis: tissue induction, hormonal regulation and cell type specification. Development 144, 1382–1398. doi: 10.1242/dev.148270, PMID: 28400434 PMC5399670

[ref73] Turco A. E. Cadena M. T. Zhang H. L. Sandhu J. K. Oakes S. R. Chathurvedula T.. (2019). A temporal and spatial map of axons in developing mouse prostate. Histochem. Cell Biol. 152, 35–45. doi: 10.1007/s00418-019-01784-6 30976911 PMC6685201

[ref74] Veltri K. Kwiecien J. M. Minet W. Fahnestock M. Bain J. R. (2005). Contribution of the distal nerve sheath to nerve and muscle preservation following denervation and sensory protection. J. Reconstr. Microsurg. 21, 57–70; Discussion 71-4. doi: 10.1055/s-2005-862783, PMID: 15672322

[ref75] Wan Y. Otsuna H. Holman H. A. Bagley B. Ito M. Lewis A. K.. (2017). Fluorender: joint freehand segmentation and visualization for many-channel fluorescence data analysis. BMC Bioinformatics 18:280. doi: 10.1186/s12859-017-1694-9, PMID: 28549411 PMC5446689

[ref76] Wegner K. A. Cadena M. T. Trevena R. Turco A. E. Gottschalk A. Halberg R. B.. (2017). An immunohistochemical identification key for cell types in adult mouse prostatic and urethral tissue sections. PLoS One 12:E0188413. doi: 10.1371/journal.pone.0188413, PMID: 29145476 PMC5690684

[ref77] Westerman R. A. Carr R. W. Delaney C. A. Morris M. J. Roberts R. G. (1993). The role of skin nociceptive afferent nerves in blister healing. Clin. Exp. Neurol. 30, 39–60, PMID: 7712628

[ref78] White C. W. Xie J. H. Ventura S. (2013). Age-related changes in the innervation of the prostate gland: implications for prostate cancer initiation and progression. Organogenesis 9, 206–215. doi: 10.4161/org.24843, PMID: 23872639 PMC3896592

[ref79] Xu Z. Hu B. Zheng G. Yu W. Yang C. Wang H.. (2024). Metformin-grafted Polycaprolactone Nanoscaffold targeting sensory nerve controlled fibroblasts reprograming to alleviate epidural fibrosis. J. Control. Release 367, 791–805. doi: 10.1016/j.jconrel.2024.02.001 38341179

[ref80] Yadav S. S. Stockert J. A. Hackert V. Yadav K. K. Tewari A. K. (2018). Intratumor heterogeneity in prostate cancer. Urol. Oncol. 36, 349–360. doi: 10.1016/j.urolonc.2018.05.008 29887240

[ref81] Yan D. Liu X. Guo S.-W. (2019). Neuropeptides substance P and calcitonin gene related peptide accelerate the development and fibrogenesis of endometriosis. Sci. Rep. 9:2698. doi: 10.1038/s41598-019-39170-w 30804432 PMC6389969

[ref82] Zahalka A. H. Arnal-Estapé A. Maryanovich M. Nakahara F. Cruz C. D. Finley L. W. S.. (2017). Adrenergic nerves activate an Angio-metabolic switch in prostate cancer. Science 358, 321–326. doi: 10.1126/science.aah5072, PMID: 29051371 PMC5783182

[ref83] Zahalka A. H. Fram E. Lin W. Mohn L. Frenette P. S. Agalliu I.. (2020). Use of Beta-blocker types and risk of incident prostate Cancer in a multiethnic population. Urol. Oncol. 38, 794.e11–794.e16. doi: 10.1016/j.urolonc.2020.03.024, PMID: 32307329

[ref84] Zahalka A. H. Frenette P. S. (2020). Nerves in cancer. Nat. Rev. Cancer 20, 143–157. doi: 10.1038/s41568-019-0237-2, PMID: 31974491 PMC7709871

[ref85] Zhang J. Harada Y. Hayashi Y. (2019). A Tlr-Cxcl1 pathway in Drg neurons induces neutrophil accumulation in the Drg and mechanical allodynia in Eae mice. Sci. Rep. 9:12003. doi: 10.1038/s41598-019-48558-7, PMID: 31427756 PMC6700073

[ref86] Zhao C. M. Hayakawa Y. Kodama Y. Muthupalani S. Westphalen C. B. Andersen G. T.. (2014). Denervation suppresses gastric tumorigenesis. Sci. Transl. Med. 6:250ra115. doi: 10.1126/scitranslmed.3009569 PMC4374618 25143365

[ref87] Zhou C. K. Check D. P. Lortet-Tieulent J. Laversanne M. Jemal A. Ferlay J.. (2016). Prostate Cancer incidence in 43 populations worldwide: an analysis of time trends overall and by age group. Int. J. Cancer 138, 1388–1400. doi: 10.1002/ijc.29894, PMID: 26488767 PMC4712103

[ref88] Zhu Y. Meerschaert K. A. Galvan-Pena S. Bin N. R. Yang D. Basu H.. (2024). A chemogenetic screen reveals that Trpv1-expressing neurons control regulatory T cells in the gut. Science 385:Eadk1679. doi: 10.1126/science.adk1679 39088603 PMC11416019

[ref89] Zhu W. Sheng D. Shao Y. Zhang Q. Peng Y. (2021). Neuronal calcitonin gene-related peptide promotes prostate tumor growth in the bone microenvironment. Peptides 135:170423. doi: 10.1016/j.peptides.2020.170423, PMID: 33086087

