# Supplementary material for: Sensory innervation in the prostate and a role for calcitonin gene-related peptide in prostatic epithelial proliferation
Source: Front Mol Neurosci. 2024 Dec 18;17:1497735. doi: 10.3389/fnmol.2024.1497735 (PMC11688385; doi:10.3389/fnmol.2024.1497735)
Supplement: Supplementary file 1 [file Table_1.DOCX]

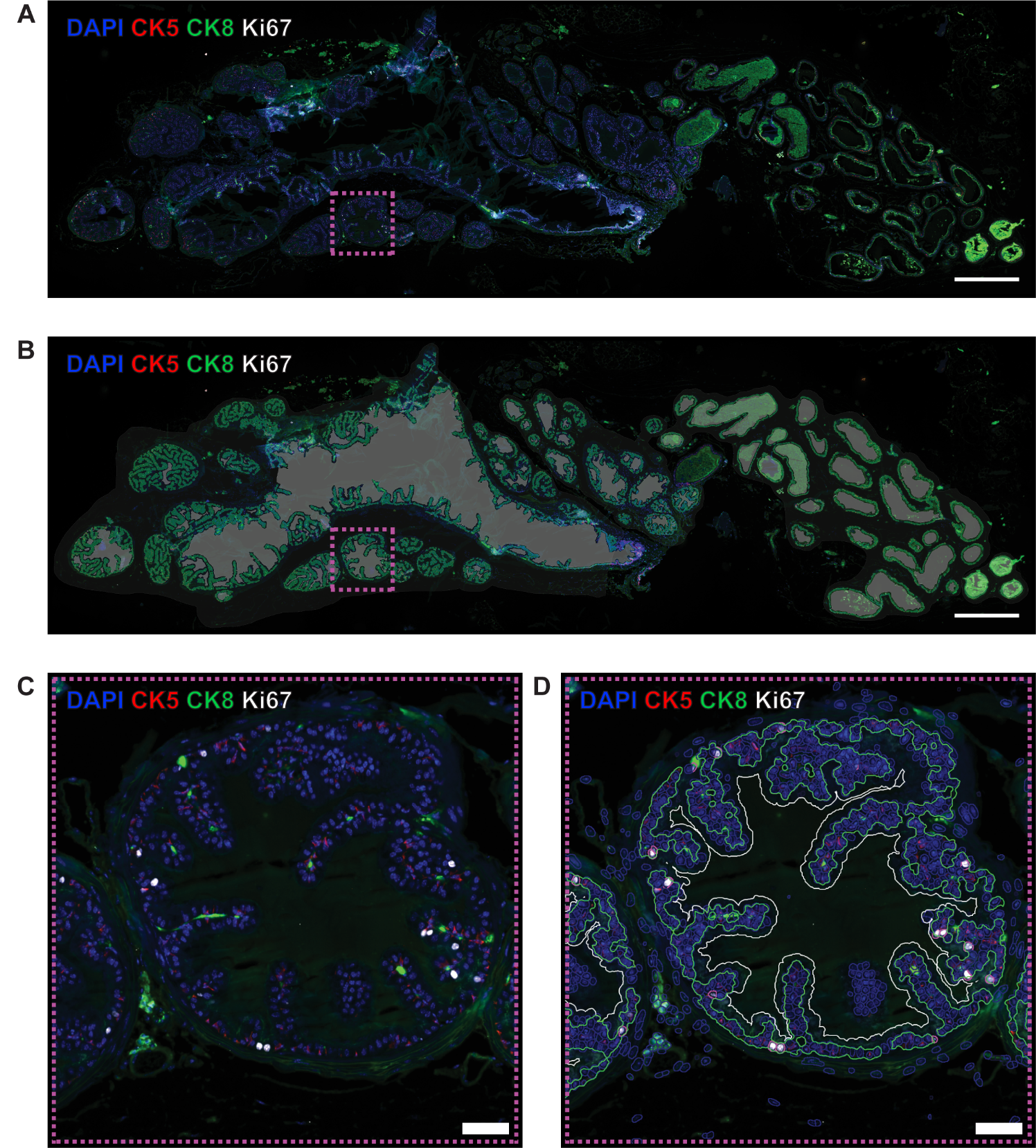
Supplementary Material

**Supplemental Figure 1.** Whole Slide Immunofluorescence Imaging of Half Mouse Prostate.

**(A)** Whole slide image showing DAPI (blue), CK5 (green), CK8 (red), and Ki67 (White) staining. **(B)** WSI with annotated ROIs denoting epithelium (green), and lumen (light gray). Magenta boxes indicate the region corresponding to insets **(C)** and **(D)**. **(C)** Higher magnification of region from **(A)** showing an anterior acini. **(D)** Annotation lines outline the epithelium (Green), lumen (white),DAPI+Ki67- (blue), and DAPI+Ki67+ (magenta) cells. Scalebars represent 500µm for **(A-B)** and 50µm for **(C-D)**.

**
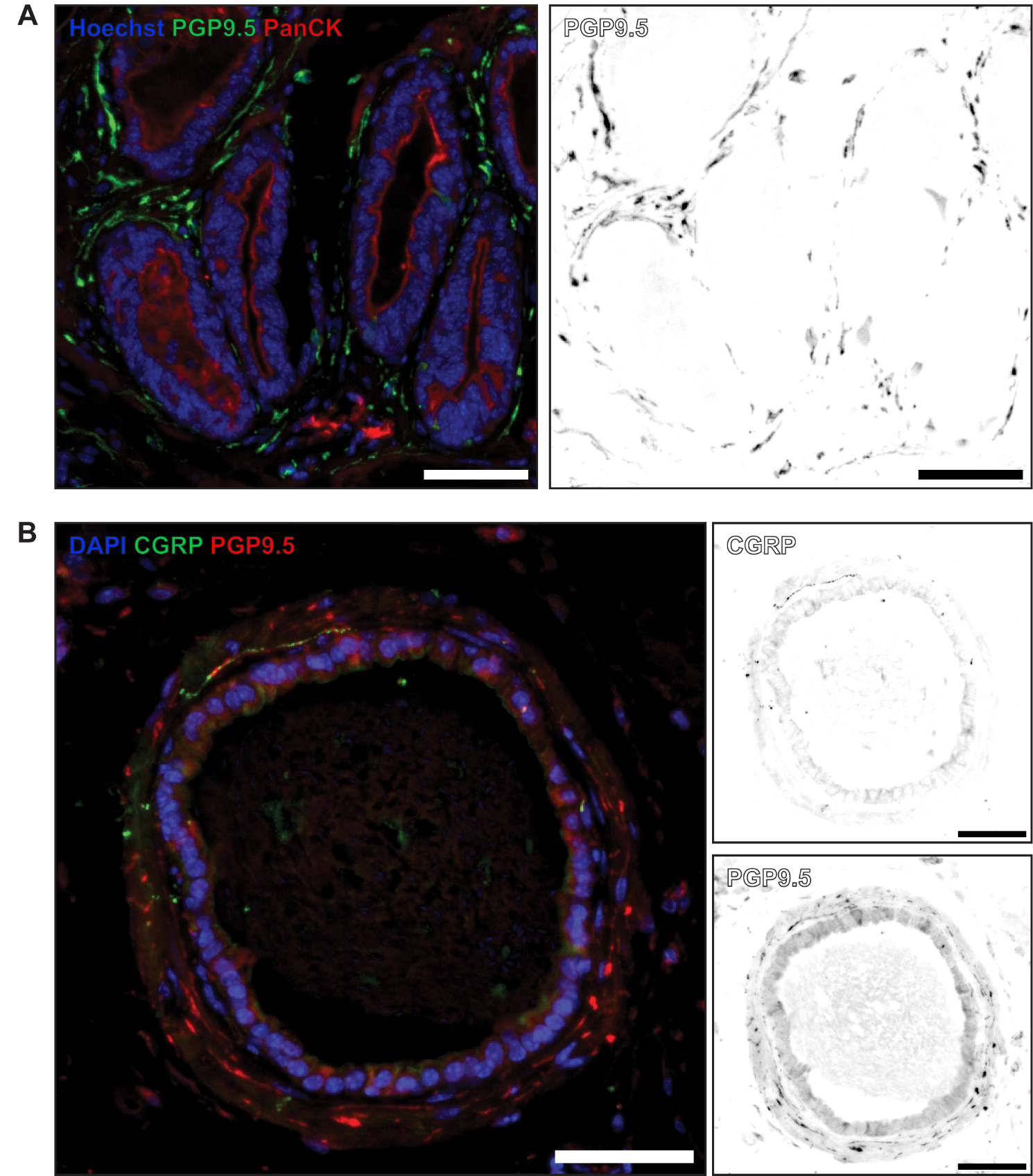
**

**Supplementary Figure 2.** Immunofluorescence imaging of mouse prostate tissue for innervating nerve fibers.

**(A)** Epifluorescence image of sectioned (5 µm) formalin-fixed paraffin-embedded mouse prostate tissue. Hoechst (blue) labels nuclei, PanCK (red) labels prostate epithelium, and PGP9.5 (green) labels peripheral nerves. Inverted grayscale image shows the PGP9.5 signal alone. **(B)** Epifluorescence image with DAPI (blue) for nuclei, CGRP (green) for peptidergic sensory nerves, and PGP9.5 (red) for peripheral nerves. Inverted grayscale images show the CGRP and PGP9.5 channels. Scalebars represent 50 µm.


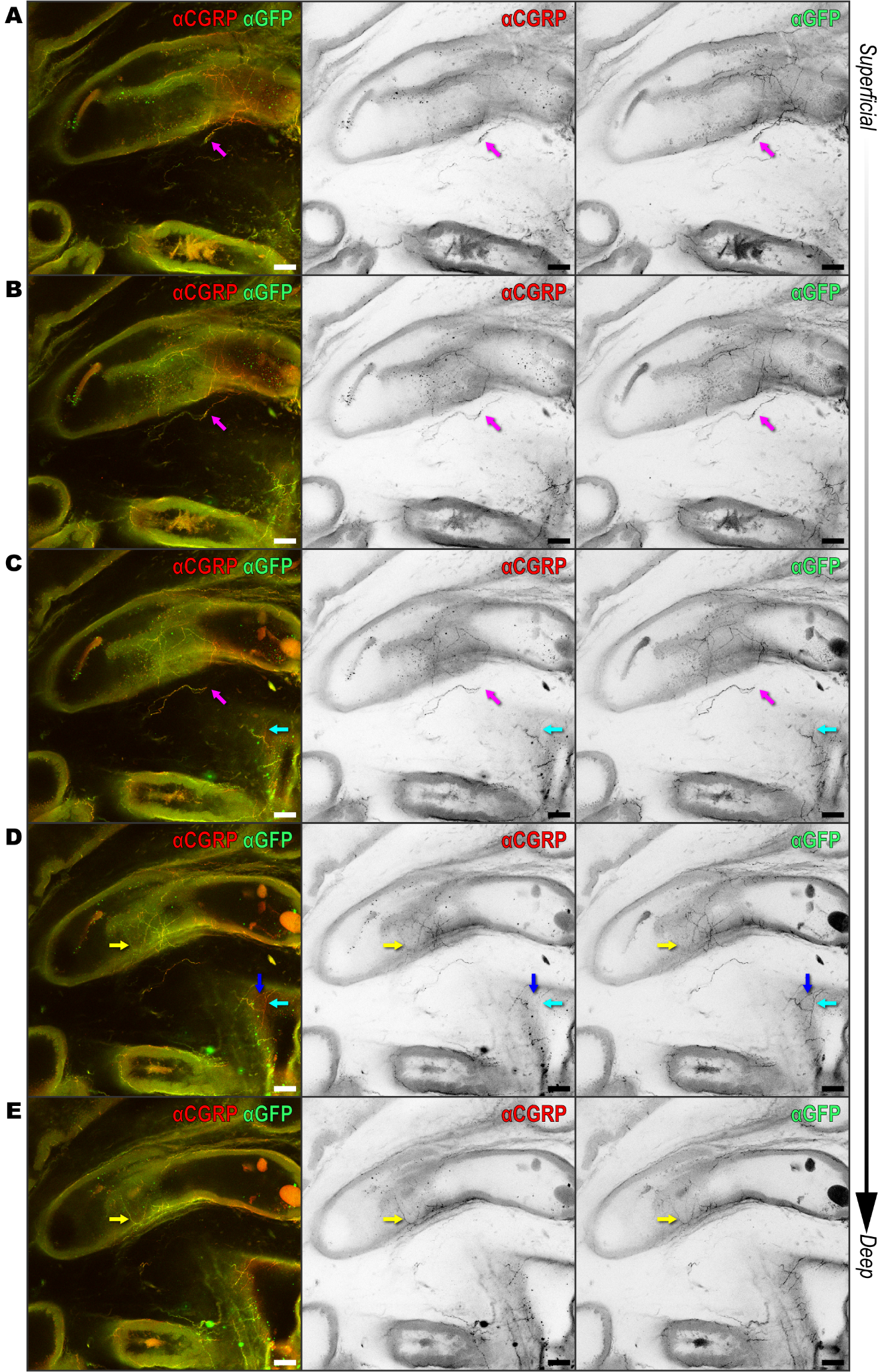


**Supplementary Figure 3.** Colocalization of CGRP and GFP signal in cleared, immunolabeled prostate tissue from a Calca^WT/GFP^ animal.

(**A-E**) 10µm maximum intensity z-projections over a 50µm z-stack from a tissue cleared mouse dorsal prostate lobe from a Calca^WT/GFP^ animal. Image triplets run from (**A**) superficial to (**E**) deep. From left to right for each triplet, composite image show immunofluorescent signal by antibody labeling for CGRP (αCGRP, red) and GFP (αGFP, green), followed by individual channels, αCGRP and αGFP, in inverted grayscale. (**A-C**) Magenta arrows denote colocalization of CGRP and GFP in a peptidergic nerve fiber traversing the prostate stroma. (**C-D**) Cyan arrows denote a peptidergic nerve fiber directly superficial to the prostate acini below with low intensity CGRP signal. (**D-E**) Yellow arrows denote another periprostatic peptidergic nerve fiber where, in the same nerve fiber, GFP signal is absent in (**D**) but present in (**E**). Blue arrows denote a nerve fiber where GFP is present, but CGRP signal appears to be absent (**D**). However, closer examination reveals faint, discrete puncta of CGRP signal that colocalizes with GFP. Scalebars represent 50 µm.
